# Highly Predictive Model for a Protective Immune Response to the A(H1N1)pdm2009 Influenza Strain after Seasonal Vaccination

**DOI:** 10.1371/journal.pone.0150812

**Published:** 2016-03-08

**Authors:** Karsten Jürchott, Axel Ronald Schulz, Cecilia Bozzetti, Dominika Pohlmann, Ulrik Stervbo, Sarah Warth, Julia Nora Mälzer, Julian Waldner, Brunhilde Schweiger, Sven Olek, Andreas Grützkau, Nina Babel, Andreas Thiel, Avidan Uriel Neumann

**Affiliations:** 1 Berlin-Brandenburg Center for Regenerative Therapies (BCRT), Charité University Hospital, Berlin, Germany; 2 Institute for Theoretical Biology, Humboldt University, Berlin, Germany; 3 Robert Koch Institute, National Reference Center for Influenza, Berlin, Germany; 4 Ivana Türbachova Laboratory for Epigentics, Epiontis GmbH, Berlin, Germany; 5 German Rheumatism Research Centre (DRFZ), Berlin, Germany; 6 Goodman Faculty of Life Sciences, Bar-Ilan University, Ramat-Gan, Israel; 7 Swiss Institute for Allergy and Asthma Research (SIAF), University of Zürich, Davos, Switzerland; Georgia State University, UNITED STATES

## Abstract

Understanding the immune response after vaccination against new influenza strains is highly important in case of an imminent influenza pandemic and for optimization of seasonal vaccination strategies in high risk population groups, especially the elderly. Models predicting the best sero-conversion response among the three strains in the seasonal vaccine were recently suggested. However, these models use a large number of variables and/or information post- vaccination. Here in an exploratory pilot study, we analyzed the baseline immune status in young (<31 years, N = 17) versus elderly (≥50 years, N = 20) donors sero-negative to the newly emerged A(H1N1)pdm09 influenza virus strain and correlated it with the serological response to that specific strain after seasonal influenza vaccination. Extensive multi-chromatic FACS analysis (36 lymphocyte sub-populations measured) was used to quantitatively assess the cellular immune status before vaccination. We identified CD4^+^ T cells, and amongst them particularly naive CD4^+^ T cells, as the best correlates for a successful A(H1N1)pdm09 immune response. Moreover, the number of influenza strains a donor was sero-negative to at baseline (NSSN) in addition to age, as expected, were important predictive factors. Age, NSSN and CD4^+^ T cell count at baseline together predicted sero-protection (HAI≥40) to A(H1N1)pdm09 with a high accuracy of 89% (p-value = 0.00002). An additional validation study (N = 43 vaccinees sero-negative to A(H1N1)pdm09) has confirmed the predictive value of age, NSSN and baseline CD4^+^ counts (accuracy = 85%, p-value = 0.0000004). Furthermore, the inclusion of donors at ages 31–50 had shown that the age predictive function is not linear with age but rather a sigmoid with a midpoint at about 50 years. Using these results we suggest a clinically relevant prediction model that gives the probability for non-protection to A(H1N1)pdm09 influenza strain after seasonal multi-valent vaccination as a continuous function of age, NSSN and baseline CD4 count.

## Introduction

Seasonal influenza is a serious infectious disease of the respiratory tract. In a typical year there are 3 to 5 million cases of severe illness, claiming up to 500.000 deaths world-wide [1]. Human influenza viruses are characterized by continuous antigenic drift and the occurrence of new variants of co-circulating influenza A and B viruses [2]. Yearly vaccination and previous infections contribute to herd immunity in the human population. However, the emergence of a new subtype of influenza virus, or the re-emergence of a subtype that has not circulated for a long time, raise the risk for a pandemic spread as it occurred in 2009 with a new A(H1N1) virus originating from pigs [3]. In the event of a newly emerging strain, rapid development of a vaccination strategy is crucial and the prediction of non-response to such vaccine is of great importance. Here we present a prediction algorithm for the response to the A(H1N1)pdm09 influenza virus strain after seasonal vaccination as a model for this situation.

Since its appearance, the A(H1N1)pdm09 virus strain was included in the annual trivalent vaccine. For the season 2011–2012 (or 2013–2014 season), A(H1N1)pdm09/California/7/2009 together with A(H3N2)/Perth/16/2009 and B/Brisbane/60/2008 (or A(H3N2)/Texas/50/2012 and B/Massachusetts/2/2012) were used as vaccine strains. The A(H3N2) and the B strains were circulating also before 2009 in the human population and accumulated small changes by antigenic drift over the time [4]. Therefore, we would expect cross-reactivity with past strains and a memory immune response after vaccination even in individuals that are sero-negative to these specific strains. In contrast, the California strain represents a new virus of the subtype A(H1N1) with other features than seasonal H1N1 viruses circulating before 2009 [5], and therefore we expect that individuals who are sero-negative to this strain have little cross-reactivity to previous strains and probably represent a primary immune response. Thus, here we attempt to predict the specific serological response to the A(H1N1)pdm09 strain following a seasonal vaccination in individuals who are sero-negative to this California strain.

Systems biology approaches and mathematical modeling are increasingly used to improve the understanding and the prediction of biological processes. Several models to predict the efficacy of influenza vaccination were published in the last years. Trtica-Majnaric et al. developed a model that included a high number of clinical variables in addition to cell populations of the immune systems [6], but with such a large number of variables the model is very difficult to use in a clinical setup. In another approach, Nayak et al. used the expansion of CD4^+^ T cells between day 0 and day 7 post-vaccination as a predictive marker [7]. A systems biology study of Nakaya et al. resulted in a high number of potential models combining the expression of 3 to 4 genes at day 0, 3 or 7 after vaccination [8]. However, using data derived after the vaccination, rather than baseline status, makes it problematic to use as a predictive model of response to vaccine. Lastly, an unbiased analysis of a high number of immune cell populations by Tsang et al. revealed a baseline signature with a predictive capacity for the post- vaccination response [9]. However, in all of these models prediction was made for the maximal sero-conversion of one out of several strains, but not for the response to a specific newly emerged strain.

Here we analyzed in a pilot study a large number of immune cell populations in individuals sero-negative for the A(H1N1)/California/7/2009 strain with respect to the post-vaccination sero-protection to that strain. We identified CD4^+^ T cells, in particular naive CD4^+^ T cells, as critical for a protective response to A(H1N1) California, but not to the A(H3N2) Perth or the influenza B Brisbane strain. Moreover, we developed a specific logistic regression model predicting sero-protection for the A(H1N1)pdm09 California strain with a high accuracy utilizing only a few baseline parameters. We had then gone further and conducted a larger validation study to particularly test the prospectively defined prediction algorithm. Based on the confirmative results from the validation study we propose a simulated model to highly accurately predict non-protection to the A(H1N1)/California/7/2009 strain.

## Materials and Methods

### Human subjects and sample collection–pilot study

This pilot study was performed at Berlin—Brandenburg Center for Regenerative Therapies, Charité—Universitätsmedizin Berlin in the fall of 2011 before seasonal influenza circulation in the community. A total of 50 donors, of which 37 (74%) vaccinees turned out to be sero-negative to the A(H1N1)/California/7/2009 strain, were recruited into the study and provided informed consent from the local ethical committee at the Charité–Universitätsmedizin. All study participants were healthy, at the age of 19–67 years and had not received any vaccine containing the 2009–2011 seasonal influenza vaccine strains, particularly the influenza vaccine A(H1N1)pdm09 strain A/California/7/2009 against S-OIV (swine-origin influenza virus). Donors who were suffering from an acute influenza like illness or other chronic illnesses were excluded. Individuals who were receiving immunomodulatory therapy, had hemoglobin value less 12 g/dl, or were pregnant were also excluded. Other exclusion criteria included known allergy to any component of the vaccine.

Overall there were 24 young donors (19–30 years) and 26 elderly donors (50–67 years). Within the young group there were 12 females and 12 males; whereas in the elderly group there were 16 females and 10 males (complete demographic data is given in S1 Table).

All donors received the trivalent inactivated influenza vaccine (TIV) Mutagrip 2011/2012 (Sanofi-Pasteur) intramuscularly by physician, including the strains: A(H1N1)/California/7/2009, A(H3N2)/Perth/16/2009 and B/Brisbane/60/2008.

Blood specimen was collected for FACS analysis immediately before vaccination at day 0. Additionally, 10 ml blood was collected in serum gel tubes for serum analysis at baseline and day 21.

### Human subjects and sample collection–validation study

A total of 71 donors were included in the validation study during the fall of 2013, of which 43 (60%) vaccinees turned out to be sero-negative to the A(H1N1)/California/7/2009 strain. The sample size for the validation study was powered for multiple testing of the 3 prospectively defined prediction algorithms that gave best results in the pilot study (NSSN, age and CD4 or CD19 or CD4 + CD19). Thus, using the prediction results from the pilot study with an allocation ratio of 3:1 protected to non-protected, power (1-beta) = 0.95 and a multiple-testing adjusted p-value = 0.0033 (starting from 0.01 and divided by 3), a sample size of 28 sero-negative donors was needed, which, using the 75% ratio of sero-negatives from pilot study, meant a total of 38 donors is to be recruited. Since we were not sure if the fraction of sero-negatives will not decrease and how many elderly donors can be recruited, we opted to recruit almost double that number (N = 71), which turned out well since the ratio of sero-negatives indeed decreased to 60%.

All donors received the trivalent inactivated influenza vaccine (TIV) Vaxigrip 2013/2014 (Sanofi-Pasteur) intramuscularly by physician. Note that in the validation study the seasonal vaccine approved for that year was somewhat different from the initial pilot study, including the strains A(H1N1)/California/7/2009, A(H3N2)/Texas/50/2012 and B/Massachusetts/2/2012. However, the same A(H1N1)/California/7/2009-pdm09 strain appeared in both vaccines and the A(H3N2) and B strains are not very different between the 2 studies, and in both cases they are not newly emerged strains. All other clinical and sampling procedures in the validation study were the same as in the pilot study. However, in the validation study a smaller number of lymphocyte sub-populations were measured.

Overall there were 22 young donors (20–30 years), 21 elderly donors (50–67 years) and 28 donors in ages 31–49 (an age group that was not included in the pilot study). Within the young group there were 16 females and 6 males; whereas in the elderly group there were 14 females and 7 males (complete demographic data is given in S2 Table).

### Ethics Statement

Both studies were approved by the ethics board of the Charité (approval numbers EA 1/175/11 and EA 1/187/13). All study participants provided written informed consent before being recruited in the study. There were no children participating in the study.

### Hemagglutination Inhibition Assay (HAI)

Influenza specific serum antibody titers were measured by a standard Hemagglutination inhibition (HAI) assay, using the seasonal 2011/2012 (or 2013/2014) vaccine strains A/California/7/2009 (A(H1N1)pdm09), A(H3N2)/Perth/16/2009 (or A(H3N2)/Texas/50/2012 in the validation study), and B/Brisbane/60/2008 (or B/Massachusetts/2/2012 in the validation study) and erythrocytes from turkey hens as previously described [10]. Pre- and post- (day 21) vaccination sera were tested simultaneously and in duplicates. Baseline sero-negativity was defined by a HAI titer below 10, while non-response and non-protection were defined as HAI below 10 and 40, respectively, at day 21. For most analyses we used non-protection, rather than non-response, since it was previously shown to be more clinically relevant.

### Isolation of peripheral blood mononuclear cells (PBMC)

PBMCs were isolated from freshly drawn heparinized whole blood in 50 ml Leucosep tubes (Greiner, Kremsmünster, Austria) filled with 14 ml Ficoll-Hypaque (LSM 1077, PAA Laboratories, Pasching, Austria) according to the manufacturer’s protocol. In brief, blood was diluted 1:1 with PBS/BSA and transferred to Leucosep tubes. Following 10 min of centrifugation at 1000 g at RT, supernatant above the leukocyte layer was removed and PBMCs were recovered by pouring them into a fresh 50 ml tube. Next, PBMCs were washed twice with PBS/BSA by centrifugation at 490 g, 10 min 4°C and 180 g, 15 min, 4°C, respectively. Finally, a sample of 10μl of PBMCs was counted with a CASY Model TTC cell counter (Roche Applied Science, Penzberg, Germany) equipped with a 150 μm capillary and with exclusion of cell debris and dead cells. Finally, PBMCs were re-suspended at a concentration of 1*10^7^ cells/ml with PBS/BSA and freshly used in subsequent assays.

### Flow cytometric analysis of absolute frequencies of leukocyte populations in peripheral blood

To determine absolute counts of the analyzed leukocyte populations in freshly drawn peripheral blood, 50 μl of heparinized whole blood was incubated for 15 minutes at room temperature with 50 μl fluorescence-labeled monoclonal antibody cocktail anti-BDCA2 (Fitc, mouse IgG1, Clone AC144 Miltenyi Biotec), anti-CD14 (VioBlue, Clone TUEK4, mouse IgG2a, Miltenyi Biotec), anti-CD19 (PEVio770, Clone LT19, mouse IgG1, Miltenyi Biotec), anti-CD3 (APC-H7, Clone SK7, mouse IgG1, BD biosciences), anti-CD4 (APC, Clone VIT4, mouse IgG2, Miltenyi Biotec), anti-CD45 (Viogreen, Clone 5B1), anti-CD56 (PE, Clone AF12/7H3, mouse IgG1, Miltenyi Biotec) and anti-CD8 (PerCp, Clone BW135/80, mouse IgG2a, Miltenyi Biotec). This staining, as well as all other staining described for other panels in the following sections were performed in the presence of Beriglobin (2μg/100μl, Sanofi-Aventis, Paris, France). Staining was stopped and erythrocytes lysed by adding 500 μl Buffer EL (Qiagen). Following 15 min incubation at 4°C, samples were immediately analyzed on a MACSQuant (Miltenyi Biotec) flow cytometer without centrifugation. The cell trigger on the instrument was set to the CD45 channel. By analyzing a defined sample up-take volume of 350 μl, MACSQuant gave direct cell counts for the labeled populations. Counts of major leukocyte populations were used for calculations of counts of any given cell subpopulation determined in other FACS panels.

### Flow cytometric analysis of innate cell subsets

To assess monocytes (classical, nonclassical and transitional) and dendritic cells (plasmacytoid, myeloid type I and II) by FACS on day 0, 5*10^6^ PBMCs of each donor were stained with a fluorescence-labeled monoclonal antibody cocktail containing anti-BDCA1 (APC, Clone AD5-8E7, mouse IgG2a, Miltenyi Biotec), anti-BDCA3 (PE, Clone AD5-14H12, mouse IgG1, Miltenyi Biotec), anti-CD20 (PerCp, Clone LT20, mouse IgG1, Miltenyi Biotec), anti-HLA-DR (Vioblue, Clone AC122, mouse IgG2a, Miltenyi Biotec), anti-CD45 (Viogreen, Clone 5B1, mouse IgG2a, Miltenyi Biotec), anti-CD3 (PerCp, Clone BW264/56, mouse IgG2a, Miltenyi Biotec), anti-BDCA2 (FITC, Clone AC144, mouse IgG1, Miltenyi Biotec), anti-CD56 (PerCpCy5.5, Clone HCD56, mouse IgG1, Biolegend), anti-CD14 (PEVio770, Clone TUEK4, mouse IgG2a, Miltenyi Biotec), anti-CD16 (APC-H7, Clone 3G8, mouse IgG1, BD Biosciences). Cells were washed once with PBS/BSA and analyzed on a MACSQuant flow cytometer. Prior acquisition, propidium iodide was added at a concentration of 0.01 μg/ml. Absolute whole blood counts of these cell populations were calculated based on events acquired in the CD14^++^ gate.

### Flow cytometric analysis of T cell phenotypes

Various T cell subsets such as naive (N), central memory (CM), effector memory (EM) and effector (Eff) CD4^+^ and CD8^+^ T cells as well as CD4^+^ CD31^+^ RTE were assessed on day 0 by a staining procedure similar to the innate panel staining, except for the incubation temperature, which was 37°C. The following antibodies were used: anti-CD8 (PEVio770, Clone BW135/80, mouse IgG2a, Miltenyi Biotec), anti-CD27 (APC, Clone M-T271, mouse IgG1, Miltenyi Biotec), anti-CD3 (Vioblue, Clone BW264/56, mouse IgG2a, Miltenyi Biotec), anti-CD31 (PE, Clone AC128, mouse IgG1, Miltenyi Biotec), anti-CCR7 (A488, Clone G043H7, mouse IgG2a, Biolegend), anti-CD45RA (efluor605, Clone HI100, mouse IgG2b, ebioscience), anti-CD45RO (efluor650, Clone UCHL1, mouse IgG2a, ebioscience), anti-CD4 (APC-H7, Clone RPA-T4, mouse IgG1, BD biosciences), anti-CD62L (PerCp-efluor710, Clone DREG-56, mouse IgG1, ebioscience) Cells were analyzed on a LSRII (BD biosciences) flow cytometer. Prior acquisition, DAPI was added at a concentration of 0.01 μg/ml. Absolute whole blood counts of these cell populations were calculated based on events acquired in the CD4 or CD8 gate, respectively.

### Flow cytometric analysis of CD4^+^ Treg phenotypes

Regulatory CD4+ T cells were assessed in 5*10^6^ PBMCs of each donor on day 0 by staining (15 min, 37°C) with the following surface antibody cocktail: anti-CD8 (PEVio770, Clone BW135/80, mouse IgG2a, Miltenyi Biotec), anti- CD31 (PE, Clone AC128, mouse IgG1, Miltenyi Biotec), anti-CD3 (PerCp, Clone BW264/56, mouse IgG2a, Miltenyi Biotec), anti-CD45RA (efluor605, Clone HI100, mouse IgG2b, ebioscience), anti-CD45RO (A700, Clone UCHL1, mouse IgG2a, biolegend), anti-CD25 (BV421, Clone BC96, mouse IgG1, biolegend), anti-CD4 (APC-H7, Clone RPA-T4, mouse IgG1, BD biosciences). After 5 min of incubation 1 μl L/D Aqua (Life technologies) was supplemented to each sample. Staining was stopped by washing PBMCs with 4 ml PBS/BSA and centrifugation (10 min, 490 g, 4°C). Then, stained cells were fixed by re-suspending them in 1 ml 1x FoxP3 fixation buffer (Ebioscience). After an incubation of 30 min in the dark at 4°C, fixed cells were washed once with PBS/BSA and spun down (10 min, 490 g, 4°C). In order to permeabilize the samples, PBS/BSA supernatant was removed and cell pellets re-suspended in 1x FoxP3 permeabilization buffer (Ebioscience). Cells were directly centrifuged (10 min, 490 g, 4°C) and supernatant carefully removed. Next, PBMCs were again re-suspended in 70 μl 1x FoxP3 permeabilization buffer and stained 30 min at RT with the following intracellular antibody cocktail: anti-FoxP3 (A488, Clone 259D/C7, mouse IgG1, BD biosciences), anti-Helios (A647, Clone 22F6, hamster IgG, BD biosciences) After a final washing step with 1x FoxP3 permeabilization buffer, cells were centrifuged (10 min, 490 g, 4°C) and pellets re-suspended in 250 μl PBS/BSA. Samples were acquired on a LSR II flow cytometer. All calculations of absolute whole blood cell counts for this panel based on the CD4 gate.

### Flow cytometric analysis of CD4^+^ Ki67^+^ and CD8^+^ Ki67^+^ T cells

Proliferating T cells were assessed on day 0 by staining 5*10^6^ PBMCs of each donor 15 min, 37°C with the following surface antibody cocktail: anti-CD4 (PerCp, Clone SK3, mouse IgG1, BD biosciences), anti-CD38 (PE, Clone IB6, mouse IgG2a, Miltenyi Biotec), anti-CD8 (V500, Clone RPA-T8, mouse IgG1, BD biosciences), anti-γδTCR (APC, Clone B1, mouse IgG1, BD biosciences), anti-CD3 (APC-H7, Clone SK7, mouse IgG1, BD biosciences), anti-CCR7 (A488, Clone G043H7, mouse IgG2a, Biolegend), anti-CD45RA (PECy7, Clone HI100, mouse IgG2b, BD biosciences). Staining was stopped by washing cells with PBS/BSA and fixating them by re-suspension in 1x BD FACS Lysing solution for 10 min at RT. Fixed cells were spun down, supernatants were discarded and 1x BD Perm 2 solution were added for permeabilization of samples. PBMCs were then washed twice with PBS/BSA and re-suspended in 100 μl PBS/BSA for intracellular staining for 30 min at RT with anti-Ki67 (V450, Clone B56, mouse IgG1, BD biosciences). Samples were measured on a MACSQuant flow cytometer. All calculations of absolute whole blood cell counts for this panel based on the CD3 gate.

### Flow cytometric analysis of B cell phenotypes

Naive and memory B cells, as well as plasmablasts were investigated on day 0 by a similar protocol as proliferating T cells, but with the following antibody cocktail: surface (15 min, RT): anti-CD14 (APCVio770, Clone TUEK4, mouse IgG2a, Miltenyi Biotec), anti-IgM (FITC, Clone MHM-88, mouse IgG1, biolegend), anti-CD19 (PEVio770, Clone LT19, mouse IgG1, Miltenyi Biotec), anti-CD27 (APC, Clone M-T271, mouse IgG1, Miltenyi Biotec), anti-CD20 (Viogreen, Clone LT20, mouse IgG1, Miltenyi Biotec), anti-CD38 (PE, Clone IB6, mouse IgG2a, Miltenyi Biotec), anti-IgD (V450, Clone IA6-2, mouse IgG2a, BD biosciences). anti-CD3 (APC-H7, Clone SK7, mouse IgG1, BD biosciences). After 5 min of incubation 1 μl of L/D Infrared (Life Technologies) was supplemented for life/dead discrimination. Intracellular staining: anti-Ki67 (PerCpCy5.5, Clone B56, mouse IgG1, BD biosciences). Samples were measured on a MACSQuant flow cytometer. All calculations of absolute whole blood cell counts for this panel based on the CD19 gate.

### Flow cytometric analysis of NK cell phenotypes

NK cells were assessed at day 0 by a similar protocol as proliferating T cells, but with the following antibody cocktail: surface (15 min, RT): anti-CD27 (APC, Clone M-T271, mouse IgG1, Miltenyi Biotec), anti-CD14 (Viogreen, Clone TUEK4, mouse IgG2a, Miltenyi Biotec), anti-CD62L (FITC, Clone 145/15, mouse IgG1, Miltenyi Biotec), anti-CD3 (V500, Clone SK7, mouse IgG1, BD biosciences), anti-CD57 (PE, Clone HCD57, mouse IgM, biolegend), anti-CD56 (BV421, Clone HCD56, mouse IgG1, biolegend), anti-CD69 (PECy7, Clone FN50, mouse IgG1, biolegend), anti-CD16 (APC-H7, Clone 3G8, mouse IgG1, BD Biosciences) After 5 min of incubation 1 μl of L/D Aqua (Life Technologies) was supplemented for life/dead discrimination. Intracellular staining: anti-Ki67 (PerCpCy5.5, Clone B56, mouse IgG1, BD biosciences). Samples were measured on a MACSQuant flow cytometer. All calculations of absolute whole blood cell counts for this panel based on the CD56 gate.

### Flow cytometric analysis of influenza specific activated CD4^+^ CD40L^+^ T cells

Influenza-specific CD40L^+^ CD4^+^ T cells were analyzed after whole blood ex-vivo stimulation with trivalent inactivated influenza vaccine (TIV) Mutagrip 2011/2012 (Sanofi-Pasteur) at day 0. For this 1 mL whole blood of each donor were incubated at 37°C with 33 μl vaccine and 1 μl anti-CD28 (BD biosciences). After 2 h Brefeldin A (10 μg/ml, Sigma-Aldrich) was supplemented to each tube. After another 4 h of incubation, 100 μl 20 nM EDTA was added to stop the stimulation. An unstimulated control (no addition of vaccine) was performed in parallel in the same way. Next, erythrocytes were lysed through addition of 10 ml Buffer EL (Quiagen), vortexing and incubation on ice for 10 min. After centrifugation, supernatants were aspirated and the remaining cell pellets were then washed once again with 4 ml Buffer EL and centrifuged. After re-suspension in PBS/BSA, cell pellets were transferred into FACS tubes, washed with again PBS/BSA and stained by a similar protocol as proliferating T cells, but with the following antibody cocktails: surface (15 min, RT): anti-CD8 (PerCp, Clone BW135/80, mouse IgG2a, Miltenyi Biotec), anti-CD20 (Viogreen, Clone LT20, mouse IgG1, Miltenyi Biotec), anti-CD14 (Viogreen, Clone TUEK4, mouse IgG2a, Miltenyi Biotec), anti-CD4 (APC-H7, Clone RPA-T4, mouse IgG1, BD biosciences). After 5 min of incubation 1 μl of L/D Aqua (Life Technologies) was supplemented for life/dead discrimination. Intracellular staining: anti-CD40L (Vioblue, Clone 5C8, mouse IgG2a, Miltenyi Biotec), anti-IFNγ (FITC, Clone 45–15, mouse IgG1, Miltenyi Biotec), anti-TNFα (PEVio770, Clone cA2, mouse IgG1, Miltenyi Biotec), anti-IL2 (APC, Clone MQ1-17H12, rat IgG2a, BD biosciences), IL4 (PE, Clone MP4-25D2, rat IgG1, Life technologies) Samples were measured on a MACSQuant flow cytometer. All measured frequencies of CD40L expressing cells were background subtracted by the matching unstimulated sample. All calculations of absolute whole blood cell counts for this panel based on the CD4 gate.

### Statistical analysis

All statistical analysis was performed using R 2.15 [11]. The pilot study concept includes two major age groups, young and elderly. However, due to the age-dependent heterogeneity of the elderly group, we decided to split this group at the median age into a middle-aged group (53–57 years, n = 16) and an old group (58–67 years, n = 10) for the analysis as indicated in the plots and results.

A two-sided unpaired Wilcoxon test was used to compare continuous variables between two groups and the Kruskal-Wallis test to compare more groups, and Fischer exact test was used to compare fraction of binary results between 2 groups. A p-value below 0.05 was defined as statistically significant. Due to the exploratory character of the pilot study, none of the pilot p-values were adjusted for multiple testing to avoid a non appropriate increase of the beta error. On the other hand, as explained above, the validation study was powered to test the 3 prospectively defined prediction algorithms that gave best results in the pilot study.

Networks of immune cell populations were created using the igraph package [12]. The colors indicate the deviations of the scaled values from the median over all donors as depicted in the graphs.

Logistic regression models were done by fitting the values to a generalized linear model based on binomial distribution. For automatic model selection the function stepAIC() from the package “MASS” was used with age, NSSN and all basic leukocyte subpopulations as start model and the Akaike information criteria for optimization [13]. Parameters of the prediction models like sensitivity, negative predictive value, positive predictive value and accuracy were determined after adjusting the specificity to 100%, capturing all non-protected donors in one group. Individual p-values for the variables in the models were determined using a two-sided t-statistic.

The one-leave-out analysis was done by taking out one donor, fitting the models to the rest of donors and predicting the one which was left out. For the drop-one analysis, each of the variables in the model was deleted separately and the new models were compared to the original model using the F test.

When transforming the age into a sigmoid shaped function we had used the logistic function: Y(X) = X_max_ / (1 + exp(-k (X-X_mid_))), where X is the actual age, Y is the transformed age, X_mid_ is the sigmoid's midpoint, X_max_ is the curve's maximum value, and k is the steepness of the curve.

## Results

### Response to A/California/7/2009 depends on age and number of strains for which the donors were sero-negative at baseline

In order to follow the serological response we determined the titer of strain-specific anti-influenza antibodies by a hemagglutination inhibition assay (HAI). We defined donors without any detectable antibodies at baseline as sero-negative (titer below 10). Donors with an antibody titer below 40 at day 21 after vaccination were considered as not sero-protected. 37 of the 50 donors in our study were sero-negative to the California strain at baseline, out of which 9 donors were still not sero-protected 21 days after vaccination.

**Fig 1:**
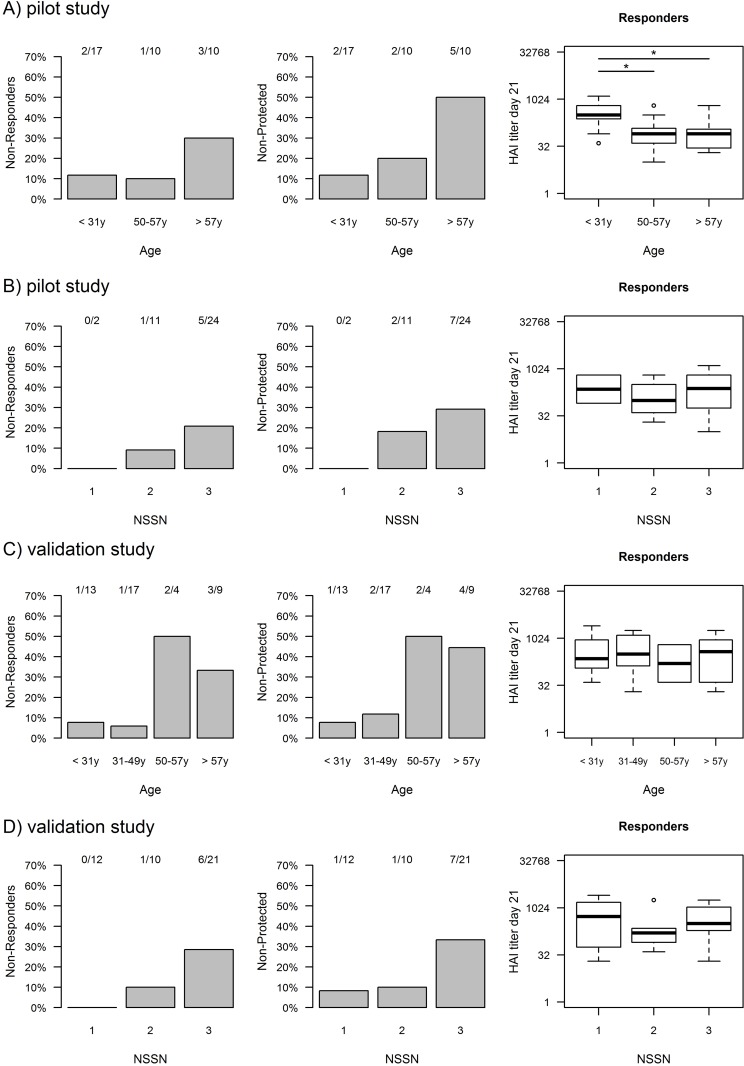
Serological response to A(H1N1)/pdm09 as function of age and number of strains that are sero-negative at baseline (NSSN) in the pilot and validation studies. A) Pilot study: Non-response (HAI<10) and non-protection (HAI<40) to A(H1N1)pdm09 at day 21 post-vaccination are higher (30% and 50%, respectively) in old (>57 years) as compared to young (<31 years) vaccinees (12% and 12%, p = NS and p = 0.04, respectively). Furthermore, HAI titers at day 21 among responders are significantly (P<0.03) lower in old donors as compared to young donors. B) Pilot study: Non-response and non-protection to A(H1N1)pdm09 are higher (21% and 29%, respectively) in donors which were sero-negative to all 3 vaccine strains at baseline (NSSN = 3) as compared to donors which were sero-negative to H1N1 but sero-positive to the other 2 strains in the vaccine (NSSN = 1, 0%, p = NS). HAI titers among responders are not related to NSSN. C) Validation study: Non-response and non-protection to A(H1N1)pdm09 at day 21 post-vaccination are validated to be higher (38% and 46%, respectively) in old (>50 years) as compared to young (<50 years) vaccinees (7% and 10%, p = 0.02 and p = 0.01, respectively). However, HAI titers among responders are not related to age in the validation study. D) Validation study: Non-response and non-protection to A(H1N1)pdm09 are higher (29% and 33%, respectively) in donors which were sero-negative to all 3 vaccine strains at baseline (NSSN = 3) as compared to donors which were sero-negative to H1N1 but sero-positive to the other 2 strains in the vaccine (NSSN = 1, 0% and 11%, p = 0.04 and p = 0.05, respectively).

We were interested to define parameters which were associated with the response. By comparing the response to the California strain within age groups, a trend for an increased risk for no response and no sero-protection in the elderly donors could be observed (Fig 1A). Whereas only 2 (12%) out of 17 young (<31 years) donors, 5 (50%) out of 10 old (>57 years) donors were not sero-protected. When taking together the <31 years and 50–57 years age groups non-protection ratio was 8.1%, which was significantly (p<0.04, Fischer exact test) lower than 50% for the >57 years group. Moreover, even within the responders the HAI titers at day 21 were significantly (p<0.03, two-sided Wilcoxon test) lower in middle aged and older donors (median = 1:80) compared to young (median = 1:320).

Since the serological baseline to other influenza virus strains might have an influence on the response to the California strain, we grouped vaccinees according to the number of influenza strains a donor was sero-negative to at baseline (NSSN) and compared their responses. We found a trend for an increase in failure to respond to A(H1N1)pdm09 in individuals with higher NSSN among the California sero-negatives (Fig 1B). However, there was no difference in the HAI titers at day 21 as function of NSSN in the responding vaccinees.

### Certain immune cell populations are specifically associated with the response to A/California/7/2009

Reportedly, the frequencies of distinct immune cell subpopulations are predictive for the maximal sero-conversion to any virus strain after vaccination [8,9]. Since the fractions of cell populations may remain unperturbed despite an overall reduction of cell numbers, we estimated the predictive capacity of immune cell counts rather than frequencies in our study.

**Fig 2:**
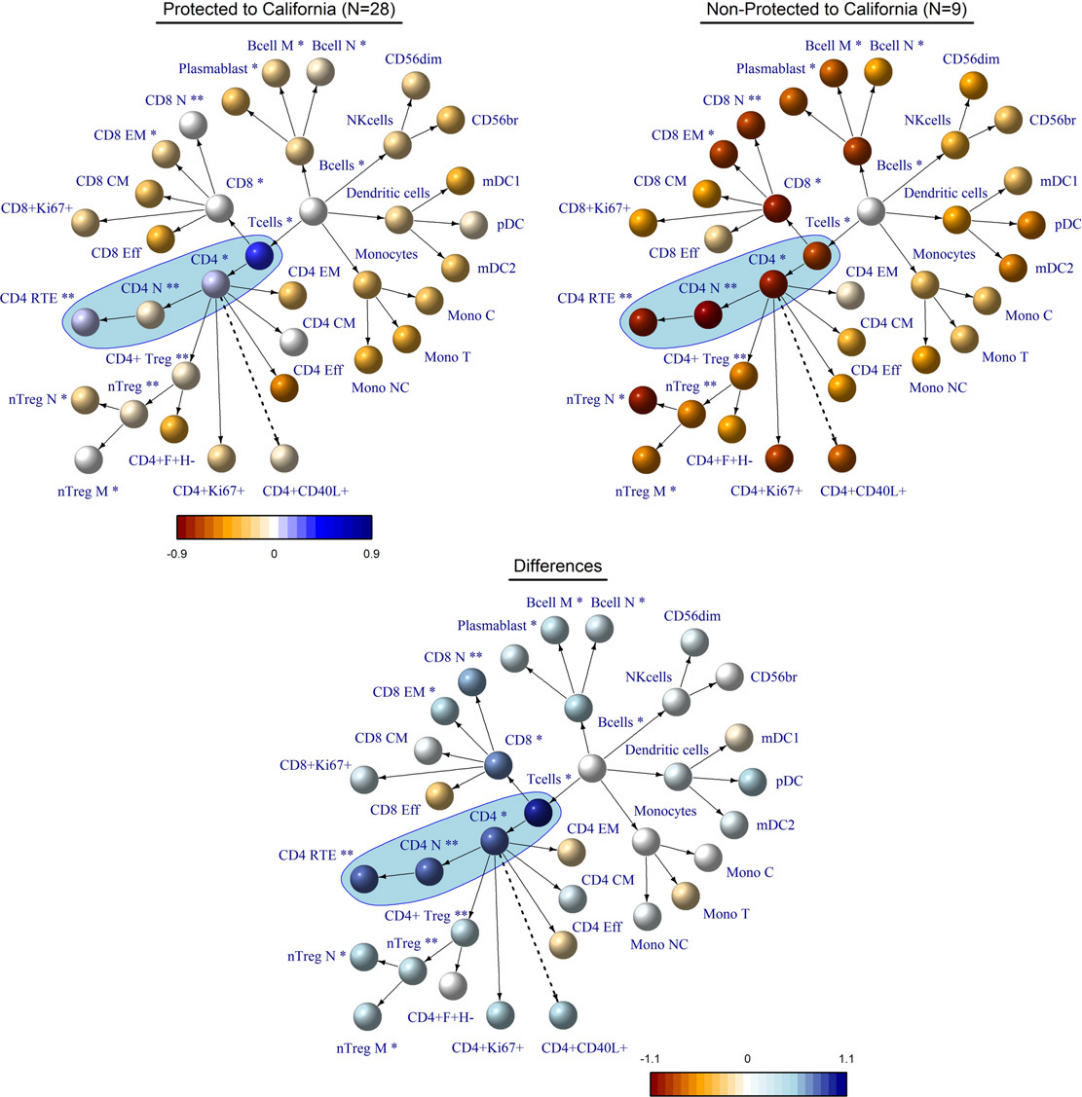
Hierarchical network representation of immune cell-subset counts at baseline with respect to A(H1N1)pdm09 protection in the pilot study. We monitored 36 immune cell subpopulations in A(H1N1)pdm09 sero-negative donors and compared donors who became either sero-protected or not at day 21 after vaccination. We observe a number of cell populations for which the counts are significantly different between protected and non-protected donors, specifically on the CD4^+^ T cell axis. The colors indicate the relative median counts of the groups. Significant differences were determined using the Wilcoxon-Test and indicated with * for p<0.05 and ** for p<0.01.

We analyzed 36 major immune cell populations by FACS for each donor prior to vaccination and compared the sero-protected versus the non-protected donor groups for the California strain (Fig 2, full data in S1 Table). Several cell populations differed significantly between the response groups (e.g. CD4^+^ T cells, CD8^+^ T cells, CD19^+^ B cells), thus suggesting good candidates for response predictors. Of particular interest, the axis of CD4^+^ T cells, CD4^+^ naïve T cells and CD4^+^ recent thymic emigrant T cells showed highly significant difference between donors protected versus non-protected to the California strain.

Analyzing the same immune cell populations in Brisbane and Perth sero-negative donors with respect to the response to these strains revealed differences in the significant immune correlates. Whereas the counts of CD8^+^ T cells and CD19^+^ B cells in Brisbane sero-negative donors and Monocytes and dendritic cells in Perth sero-negative donors were significantly different between the protected and non-protected groups, to each strain correspondingly, no significant deviations in the CD4^+^ T cells and its subsets could be observed (S1 and S2 Figs).

Due to the predictive value of age and NSSN, we had tested the association of the various immune cell populations with these variables (S3 and S4 Figs). While CD8^+^ T cell count is lower in older individuals and CD14^+^ Monocyte count is higher in older individuals compared to the young group, none was significantly associated with NSSN.

### Baseline CD4^+^ T cell count is best predictor for protection against A/ California/7/2009

**Fig 3:**
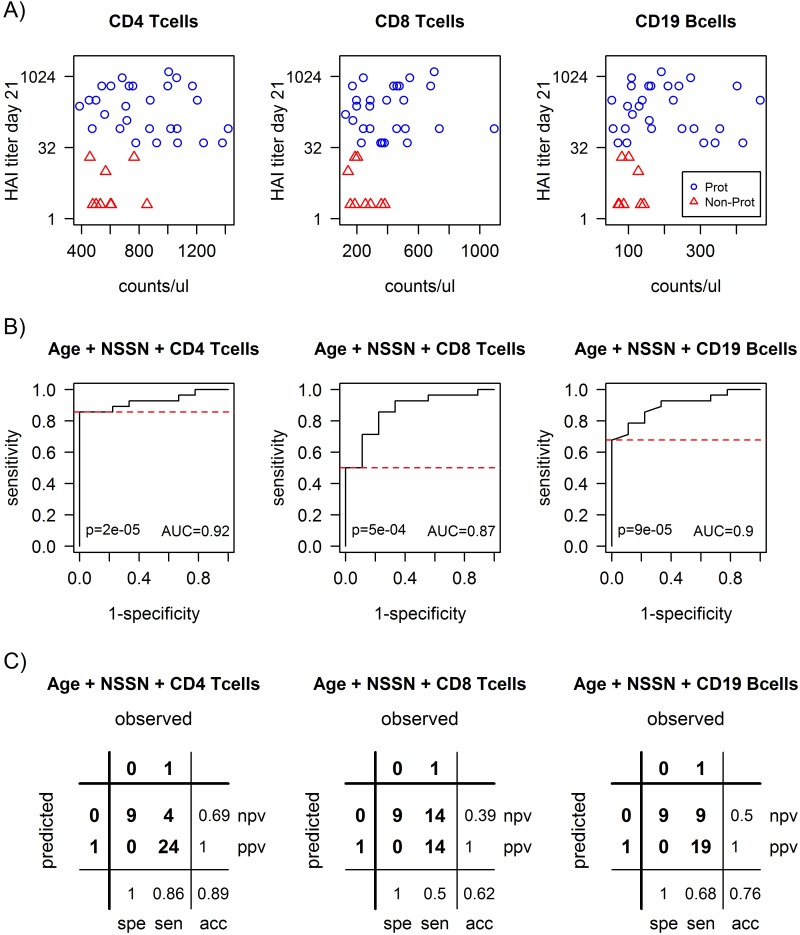
Prediction of serological response to A(H1N1)pdm09 as function of counts of major lymphocyte sub-populations in the pilot study. A) H1N1 sero-negative individuals that were sero-protected (blue circles) to H1N1 at day 21 post-vaccination had higher counts of CD4^+^ T cells, CD8^+^ T cells or B cells (CD19) at baseline as compared to non-protected individuals (red triangles, P<0.05). However, there is no continuous correlation between the various cell counts and the HAI titer. B) Logistic regression shows that a model combining baseline CD4^+^ T cell counts with age and NSSN, is the best predictor of sero-protection, with a high ROC-AUC = 0.92 and significant p-value = 0.00002. Similar models for CD8^+^ T cells or B cells (CD19^+^) give reasonable albeit lower prediction values. C) The combination of CD4^+^ T cell counts, NSSN and age gives a highly accurate 89% prediction of non-protection (when selecting for a specificity = 100%, or positive predictive value ppv = 100%, in order to capture all non-responders), with a sensitivity of sen = 86% and negative predictive value npv = 69%. The other lymphocyte sub-populations counts give less accurate predictions.

Based on combination of each of the various predictive baseline immune cell population counts, together with age and NSSN, we tested various logistic regression models to predict protection against the A(H1N1)/California/7/2009 strain in California sero-negative donors (Fig 3 and S5 Fig). The logistic regression model which includes the CD4^+^ T cells showed the best prediction with an accuracy of 89%, a sensitivity of 86% along with a specificity of 100% (p = 0.00002).

None of the models with other cell sub-populations had a better predictive capacity regarding the accuracy compared to the model with age, NSSN and CD4^+^ T cells. The next best predictive model included Age, NSSN and CD19^+^ B cells with an accuracy of 76%. We further tested the total amount of lymphocytes or leukocytes as predictive markers for protection against the California strain, but their accuracy and significance were lower than that for CD4^+^ T cells (S6 Fig).

In order to test for robustness we performed a one-leave-out cross validation of all models, with the best result again for Age together with NSSN and CD4^+^ T cells (p = 0.0006).

Importantly, CD4^+^ T cells and age of donors were significantly independent in the logistic regression model (p<0.03 and p<0.02, respectively). Moreover, a drop one analysis revealed, that all three variables including NSSN significantly influenced the model prediction (F test, p<0.05). In fact, a model including only age together with NSSN (S6 Fig), or each of the variables alone, gave poor predictive results.

### Baseline naive CD4^+^ T cell count is associated with protection

**Fig 4:**
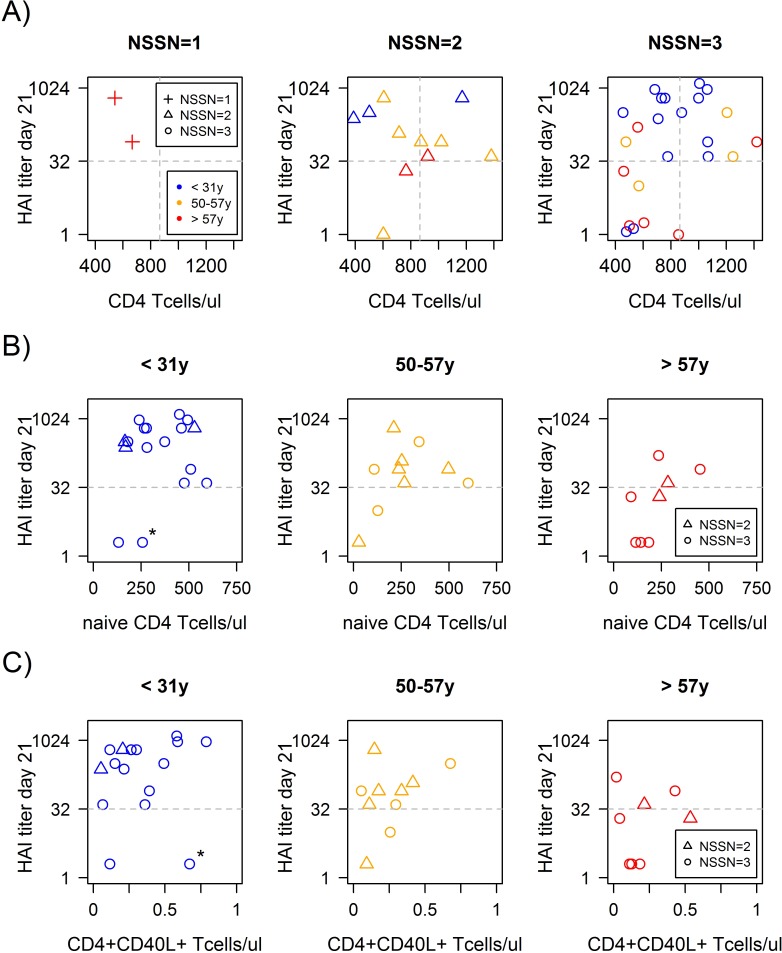
Multi-factorial association of serological response to the A(H1N1)pdm09 influenza strain as function of age, NSSN and total, naïve and influenza specific activated CD4^+^ T cells in the pilot study. A) High CD4^+^ T cell counts give rise to sero-protection (HAI>40) irrespective of age or NSSN. Age still plays a role in vaccinees with NSSN = 3 and low CD4^+^ T cell counts, where 80% of old (red circles) versus only 20% of young (blue circles) are non-protected (p = 0.01). The same trend (p = NS) is also seen for NSSN = 2 (triangles) with low CD4^+^ T cell counts, albeit with better response than NSSN = 3. The only 2 donors with NSSN = 1 are sero-protected even if they are old and have low CD4^+^ T cell counts. B) Naive CD4^+^ T cell counts show a trend (p = NS) for a positive association with serological response in all age groups (NSSN = 2–3). C) Influenza specific activated CD4^+^CD40L^+^ T cell counts are not associated with serological response in any of the age groups (NSSN = 2–3).

All donors with high CD4^+^ T cell levels have very small probability for being non-protected after vaccination, independently of age and NSSN (Fig 4A). However, for the donor group with low CD4^+^ T cell values, the situation is more complex. To find out why some of the members of this group responded whereas other failed, we analyzed CD4^+^ T cell subpopulations in more detail.

As expected, we found higher naive CD4^+^ T cell counts in the young than in the older age groups (Kruskal-Wallis test, p<0.01). Moreover, naive CD4^+^ T cells counts show a potential for prediction of response irrespective of age (Fig 4B). A trend for positive association between baseline naive CD4^+^ T cells count and response to A(H1N1)pdm09 was observed in the middle aged and the old groups. Of the two young non-protected donors, one had low naive CD4^+^ T cell count and the other (marked with asterisk in Fig 4B), while having somewhat higher naive CD4^+^ T cell count, had the lowest values in the study for Monocytes, plasmablasts and other leukocyte subpopulations, suggesting that his immune system was probably compromised.

However, a prediction model using age and NSSN together with naive CD4^+^ T cell count at baseline, although highly predictive, did not give better prediction than using total CD4^+^ T cell count at baseline (S7 Fig). A further subdivision of the naive CD4^+^ T cell population into CD31^+^ recent thymic emigrants (RTE) and CD31^-^ non-RTE fractions did not result in an improvement of the prediction, although both are predictive of protection.

### Baseline influenza-specific activated CD4^+^ T cell counts are not associated with protection

One might presume that the baseline immune memory capacity against the vaccine antigens would be an important predictor of the response to the vaccine. We had therefore measured activated CD4^+^ CD40L^+^ T cell counts after stimulation with the 3 influenza strains in the vaccine. However, the baseline counts of the influenza specific activated CD4^+^ CD40L^+^ T cells were not associated with protection against the California strain (Fig 4C) irrespective of age and NSSN. Furthermore, a model including the influenza vaccine specific activated CD4^+^ CD40L^+^ T cells together with age and NSSN (S5 Fig) gave poor predictive results for protection against the California strain.

### Combinations of 2–3 immune sub-populations baseline counts

In order to try and further improve the predictive model, we ran an automatic backward selection starting with all immune cell subpopulations, age and NSSN, allowing for testing combinations of 2–3 immune sub-population baseline counts, possibly together with age and NSSN, as predictors for protection to the California strain. Using the Akaike information criteria for optimization, a model including CD4^+^ T cells as well as CD19^+^ B cells together with age and NSSN was the most significant (p = 0.00002) with an accuracy of 92% (S8 Fig). All California non-protected donors were characterized by combined low numbers of CD4^+^ T cells and CD19^+^ B cells. However, the counts of CD4^+^ T cells and CD19^+^ B cells are highly correlated to each other (p<2*10^−9^, R = 0.82) and thus the robustness of this model was not very high. All other multiple combinations of baseline immune sub-populations counts gave predictive models that were not significant when tested by one-leave-out procedure.

### Validation study to test the prospectively defined predictive model from the pilot study

In order to validate the results obtained in the pilot study we had conducted an additional validation study powered to prospectively test the 3 best prediction models found above. Thus, in the fall of 2013 we had recruited 71 healthy donors that were vaccinated with the trivalent inactivated influenza vaccine Vaxigrip 2013/2014, including the strains A(H1N1)/California/7/2009, A(H3N2)/Texas/50/2012 and B/Massachusetts/2/2012. The same A(H1N1)/California/7/2009-pdm09 strain appeared in both studies and the A(H3N2) and B strains are not very different between the 2 studies, and in both cases they are not newly emerged strains. Interestingly, only 43 (60.6%) of the vaccinees in the validation study were found to be sero-negative to the H1N1 California strain, as compared to 74% of vaccinees in the fall of 2011, although in both cases one of the inclusion criteria was no previous vaccination against this strain. 9 of the 43 sero-negatives (21%) were still not sero-protected 21 days after vaccination.

Both age and NSSN were validated to be significant predictors of protection to the California strain among donors sero-negative to that strain at baseline (Fig 1C and 1D). Only 7% (or 10%) of sero-negative donors below the age of 50 were non-responders (or non-protected), as compared to 38% (or 46%) of those above 50 years (p = 0.02 and p = 0.01 respectively). Similarly, only 5% (or 9%) of sero-negative donors with NSSN = 1 or 2 were non-responders (or non-protected), as compared to 29% (or 33%) of those with NSSN = 3 (p = 0.05). However, we did not find in the validation study significant differences in the HAI titers at day 21 among the responders as function of age or NSSN.

**Fig 5:**
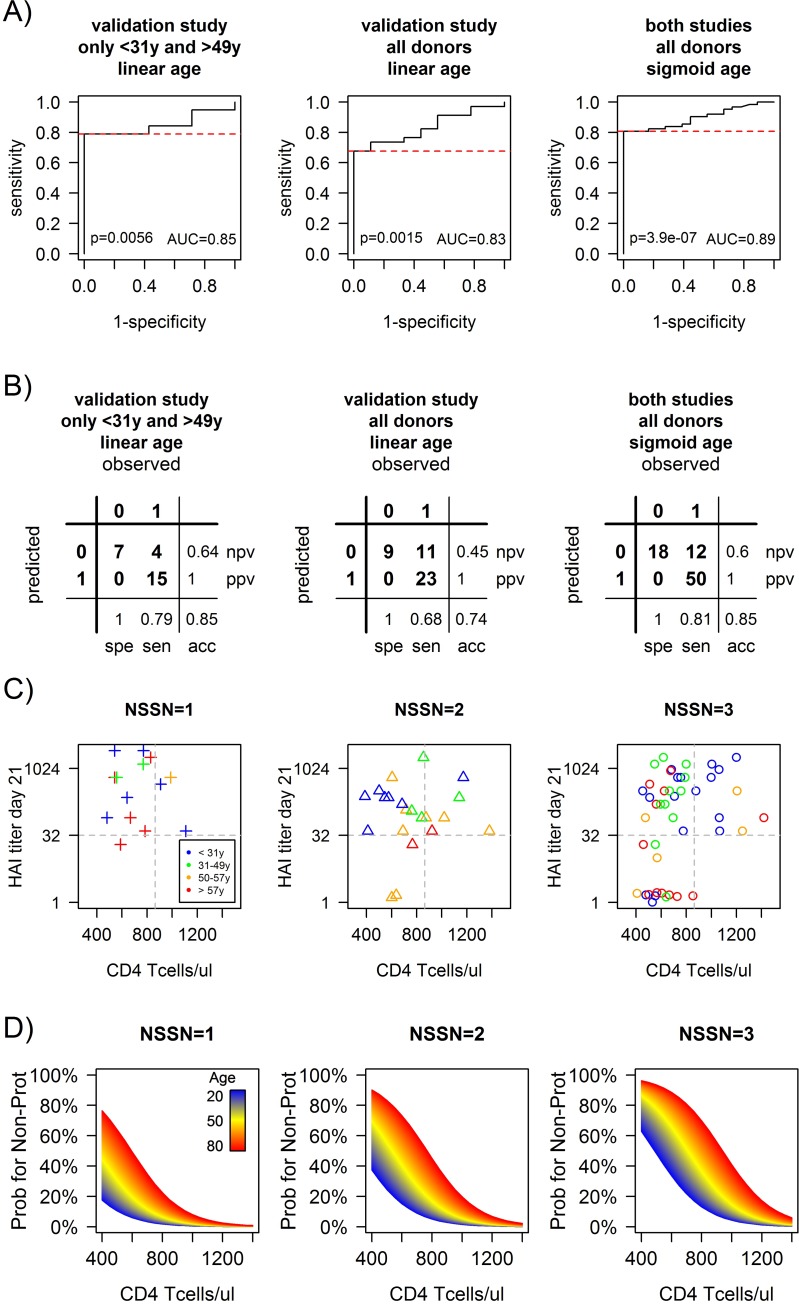
Prediction of non-protection to the A(H1N1)pdm09 influenza strain as function of the combination of age, NSSN and CD4^+^ T cells after the validation study. The logistic regression model combining baseline CD4^+^ T cell counts with age and NSSN is validated with a high ROC-AUC = 0.85, significant p-value = 0.0056 and high accuracy of 85% for the same age groups (<31 and >49 years) as in the pilot study (left panels in A and B). However, the addition of the middle age group (31–49 years) in the validation study somewhat reduces the accuracy of the prediction when using age as a linear function (center panels in A and B), because donors with these ages respond rather like the younger donors. Transformation of age to a sigmoid based function (with a midpoint age of 50 years) gives the best prediction with accuracy 85% and a highly significant p-value = 0.0000004 when combining both studies (right panels in A and B). The multi-factorial risk profile for non-protection (HAI<40) to the A(H1N1)pdm09 influenza strain is clearly seen (C) when combining the sero-negative vaccinees from both studies (N = 80). Donors with high baseline CD4^+^ T cell counts (>860 cells/μL) are all protected (p = 0.02 for NSSN = 3), as well as young (<50 years) donors with low CD4^+^ counts but NSSN = 1–2. Non-protection is only observed for old donors with low CD4^+^ counts (20%, 50% and 64% for NSSN = 1, 2 and 3 respectively) and for young donors with low CD4^+^ counts and NSSN = 3 (24%). Lastly, a prediction model (D) for the probability of non-protection to the California H1N1 strain is obtained by simulating the continuous contribution of age (after logistic function transformation from 20 years young in blue to 80 years old in red), NSSN and baseline CD4^+^ T cell counts, where the combined effect of the 3 variables can be clearly seen.

Furthermore, when considering the validation study donors with the same age groups as in the pilot study (N = 13 with <31 years and N = 13 with >49 years), the best logistic regression prediction model from the pilot study, which includes age, NSSN and the CD4^+^ T cell count at baseline, was validated with a high accuracy of 85% (similar to the pilot study) and a p-value of p = 0.0056, which is significant even considering the multiple testing of the 3 prospectively defined models (left panels in Fig 5A and 5B). However, the models including age and NSSN together with CD19^+^ B cell count at baseline, or together with both CD4^+^ T cells and B cells, did not come out as significantly predictive in the validation study.

Interestingly, when including all the donors in the validation study (i.e. including also N = 17 with ages 31–49 years) the model including age, NSSN and CD4^+^ baseline count still came out as significantly predictive (p = 0.0015) albeit with a somewhat lower accuracy of 74% (middle panels in Fig 5A and 5B). Inspection of the data has indicated that vaccinees of the ages 31–49 years tend to behave more like the younger rather the older donors. Thus, we had tested a sigmoid shaped transformation function for the age instead of the linear age initially used. Indeed, we had found that a logistic age function (see methods) with a midpoint value of 50 years, maximum value of 60 years and a steepness of 0.2 gives the best predictive results when including all sero-negative donors from both studies (N = 80), with an accuracy of 85% and a highly significant p-value of 0.0000004 (right panels in Fig 5A and 5B).

### Model simulation of the multi-factorial risk profiles

Combining the results from both studies (N = 80 donors sero-negative for the California strain) we identified different risk profiles with a multi-factorial dependence on age, NSSN and baseline CD4^+^ T cell counts (Fig 5C). All donors with high baseline CD4^+^ counts (>860 cells/μl) are protected against the California strain irrespective of age and NSSN (100% versus 70% for high versus low baseline CD4^+^, p = 0.009). Among donors with low baseline CD4^+^, young vaccines (<50 years) have significantly higher probability for protection (86%) compared to old vaccines (48%, p = 0.002). Also, among donors with low baseline CD4^+^, the number of strains one is sero-negative for at baseline has an impact on the probability for protection (91%, 80% and 60% for NSSN = 1, 2 and 3 respectively, p = 0.05).

Young donors (<50 years) with low CD4^+^ T cell counts (<860 cells/μl) that have NSSN = 1 or NSSN = 2 are also all protected, although those that are sero-negative for all strains at baseline (NSSN = 3) still have a good chance (76%, p = 0.05) to be protected. In contrast, old donors (≥50 years) with low CD4^+^ T cell counts (<860 cells/μl) have a good chance to achieve sero-protection only if they are sero-negative for only the California strain (NSSN = 1: 80%), as compared to those who are sero-negative for all three strains in the vaccine (NSSN = 3) that have a low probability for protection (36%).

Based on these observations and using the logistic regression model with the sigmoid age function, NSSN and CD4^+^ T cell baseline count we simulated the probability for non-protection as a continuous function of age and CD4^+^ T cell baseline count for the different NSSN status (Fig 5D). Using this simulation it is possible to estimate the probability of non-protection to the H1N1 California strain for any arbitrary combination of these 3 variables.

## Discussion

We have conducted an extensive exploratory study followed by a validation study to predict protection against the pandemic influenza A(H1N1)pdm09 California strain by the immune status pre-vaccination. We have focused on predicting the serological immune response (measured by hemagglutination inhibition assay, HAI) to the newly emerged influenza strain A/California/7/2009 rather than the combined response to all three strains in the seasonal tri-valent influenza vaccine. The other strains used in the seasonal vaccinations in our study were the H3N2 strains (A/Perth/16/2009 or A/Texas/50/2012) and the influenza B strains (B/Brisbane/60/2008 or B/Massachusetts/2/2012) that were circulating, either themselves or in similar strains, in the human population for a longer time. Indeed, we observed that the immune cells sub-populations that show a significant difference between responders and non-responders, are not the same for each of those antigens. In particular, total CD4^+^ T cell counts at baseline together with age and the number of influenza strains that an individual was sero-negative to at baseline (NSSN) were highly predictive for the response against the new H1N1 California strain in vaccinees that were sero-negative to that strain at baseline.

Since naive CD4^+^ T cells are dispensable in a memory response, our observations that naïve CD4^+^ T cell counts at baseline, rather than influenza specific (for all 3 vaccine strains together) CD4^+^CD40L^+^ activated T cell counts at baseline, were highly associated with the serological response to the H1N1 California strain, indicate that the CD4^+^ T cell immune response in sero-negative donors is indeed more a primary-like, than a memory-like, immune response against the newly emerged strain. A critical part of the primary immune response is the stimulation of virus-specific B cells by specific CD4^+^ T cells, recruited from the naive pool. The chance to select CD4^+^ T cells with a high binding capacity will increase with the number of available naive CD4^+^ T cells. These results are in accordance with a recent publication demonstrating that A(H1N1)pdm09 virus induced two types of CD4^+^ T cell responses after both infection or vaccination [14]. One type of CD4^+^ T cells recognized conserved regions of the hemagglutinin and was recruited from the memory pool. The major immune response was in contrast directed against unique features of the virus strain and the corresponding CD4^+^ T cells were selected from the naive CD4^+^ T cell pool. The CD4^+^ T cell expansion was recently also described to be predictive for the neutralizing antibody response to a monovalent 2009 A(H1N1)pdm09 vaccine [7]. Moreover, similar to our study, low CD4^+^ T cell counts in well controlled HIV-infected patients resulted in poor protection rate after receiving a A(H1N1)pdm09 vaccine [15]. It will be interesting to verify if the kinetics, rather than the baseline level, of influenza specific CD4^+^CD40L^+^ activated T cells is correlated with the H1N1 serological response.

Remarkably, the response to the new California strain was strongly depending also on the number of strains for which each donor was sero-negative to at baseline (NSSN) among donors sero-negative to the California strain. Lower protection rates were found in donors that were sero-negative for all 3 strains (NSSN = 3) as compared to higher protection in donors that were sero-positive for either Perth or Brisbane (NSSN = 2) and in donors that were sero-positive to both Perth and Brisbane (NSSN = 1). Although the California 2009 H1N1 strain is a newly emerged influenza virus with a swine origin, higher number of strains with sero-positivity (lower NSSN) may be a marker for higher cross-reactivity to pre-circulating strains that could play a role in a more rapid generation of hemagglutination antibodies against the newly emerged H1N1 virus. Alternatively, this could be explained by the necessity to divide limited resources, which are critical for a successful immune response, between a primary immune responses to 3 strains in the NSSN = 3 donors, as compared to the donors which are already sero-positive for some of the strains and may raise a memory response to them.

Age was still a critical factor in the prediction of non-protection to A(H1N1)pdm09. Comparing the protection rates for donors at ages 35–50 with those younger and older, shows that the predictive function of age is not linear but rather sigmoid with a midpoint at about 50 years. The availability of naive CD4^+^ T cells may account for part of the age effect, since we observed that naive CD4^+^ T cell count was significantly higher in the young donor group as compared to old. However, low naive CD4^+^ T cell count may increase the probability for non-protection irrespective of age, but age still plays a role among vaccinees with low counts of naive CD4^+^ T cells (Fig 4B). Thus, other age related factors still need to be elucidated in order to completely understand the age effect.

The correlation between age and counts of various immune cell populations is also important to understand the selection of independent predictors in the multi-variate logistic regression model. A strong association between younger age and higher CD4^+^ naive T cell, as well as CD4^+^ regulatory T cell, counts is well documented in the literature and was also found in our study (S1 Table). Thus, although both CD4^+^ naive and CD4^+^ Treg variables were significantly associated with successful immune response against A(H1N1)pdm09 (Fig 2), they were not selected as significant independent predictors in the logistic regression model due to their strong interaction with age. On the other hand, total CD4^+^ T cell counts were not significantly associated with age, or with NSSN, and therefore the CD4^+^ baseline count variable emerges as a significant independent predictor even when combined with age and NSSN.

Interestingly, both sub-populations of CD4^+^ naive T cells, recent thymic emigrants (RTE, CD31^+^) and non-RTE (CD31^-^), contributed similarly to the prediction of non-protection to H1N1. The reason for this might be that indeed RTEs are the important factor, but the association between CD4 Naive count and age creates a confluence also with the non-RTE population.

In conclusion, we have extensively tested a large number of immune markers before vaccination with seasonal influenza vaccine and found that a surprisingly simple model predicts the success of serological response to the newly emerged influenza A(H1N1)pdm09 virus A/California/7/2009. Combining age, number of sero-negative strains at baseline (NSSN) and baseline CD4^+^ T cell counts allows predicting protection to A(H1N1)pdm09 with a highly statistical significance and an accuracy of 85%, in particular capturing all the non-protected vaccinees. The rigorous testing methodology we have undertaken, including one-leave-out and drop-one procedures to select the best predictive models during the exploratory pilot study, as well as the followup validation study, which was conducted with power for multiple testing of the 3 prospectively defined best models from the first phase, ensures the robustness of our results. The combined population of both studies, with N = 80 vaccinees sero-negatives to the H1N1 California strain, clearly elucidates the multi-factorial effect of the 3 predictors in the model. Finally, the simulation predictive model we suggest is simple enough and accurate enough to allow its use in clinical settings. It would be interesting to conduct similar analysis of immune response to other newly emerging viral strains to test if this prediction model is specific for the A(H1N1)/California/7/2009 pandemic strain or more generic.

## Supporting Information

S1 Fig Hierarchical network representation of immune cell-subset counts at baseline with respect to the Brisbane strain protection in the pilot study. Overall, 36 immune cell subsets at baseline were analyzed and compared in Brisbane sero-negative donors with or without sero-protection for the Brisbane strain after vaccination using the Wilcoxon test. P-values below 0.05 and below 0.01 were indicated with one or two asterisks, respectively.
(TIF)

S2 Fig Hierarchical network representation of immune cell-subset counts at baseline with respect to the Perth strain protection in the pilot study. Overall, 36 immune cell subsets at baseline were analyzed and compared in Perth sero-negative donors with or without sero-protection for the Perth strain after vaccination using the Wilcoxon test. P-values below 0.05 and below 0.01 were indicated with one or two asterisks, respectively.
(TIF)

S3 Fig Baseline immune cell counts as a function of age in the pilot study. The counts of CD8+ T cells and Monocytes are significantly different between young and old donors (two-sided Wilcoxon test, p<0.05).
(TIF)

S4 Fig Baseline immune cell counts as a function of NSSN in the pilot study. No significant differences related to the number of sero-negative strains were observed in A(H1N1)pdm09 sero-negative donors.
(TIF)

S5 Fig Logistic regression models with monocytes (CD14^+^), natural killer (CD56^+^ NK) cells, dendritic cells (pDCs), plasmablasts and influenza specific activated (CD4^+^CD40L^+^) cells in the pilot study. Plasmablasts counts are significantly (p = 0.03) different between H1N1 protected and non-protected vaccinees, while the other sub-populations in this figure show no significant association with protection on their own. The prediction of serological response using multi-variate logistic regression including baseline immune cell populations, age and NSSN is presented. Even if significant, the results for these cell populations are not as good as the CD4^+^ T cell model. In particular of interest that the model using baseline counts of specifically activated cells (CD4^+^CD40L^+^) sorted after stimulation with the 3 influenza strains in the vaccine does not give a good prediction.
(TIF)

S6 Fig Logistic regression models with age only, CD4^+^ T cells, Lymphocytes and Leukocytes in the pilot study. Age, CD4 T-cell and lymphocyte counts are significantly (p = 0.01, p = 0.04 and p = 0.05, respectively) different between H1N1 protected and non-protected vaccinees, while leukocytes show no significant association with protection on their own. Leukocytes and lymphocytes multivariate logistic regression models (including age and NSSN) are not as good as the CD4^+^ T cell multivariate logistic regression model. Due to limited availability of leukocyte data this analysis was performed in only N = 31 California sero-negative donors. Also the model using age and NSSN alone, without CD4^+^ T cell counts, does not give a good prediction.
(TIF)

S7 Fig Logistic regression models with CD4^+^ T cells, naive CD4^+^ T cells, naive CD4^+^CD31^+^ recent thymic emigrants T cells (RTE) and naive CD4^+^CD31^-^ non-RTE T cells in the pilot study. Counts of CD4 total T-cells, CD4 Naive T-cells, CD4 Naïve RTE T-cells and CD4 Naïve non-RTE T-cells are all significantly (p = 0.02, p = 0.002, p = 0.009 and p = 0.005, respectively) different between H1N1 protected and non-protected vaccinees. Prediction of serological response using multi-variate logistic regression including baseline immune cell populations, age and NSSN is presented. The prediction using the naive CD4^+^ T cells, RTE or non-RTE cells, is significant albeit less accurate than using the total CD4^+^ T cells.
(TIF)

S8 Fig Prediction of non-protection to A(H1N1)pdm09 strain as function of the combination of age, NSSN, CD4^+^ T cells and B cells in the pilot study. Using multi-variate logistic regression shows that the combination of age, number of sero-negative strains (NSSN), CD4^+^ T-cell counts and B-cell counts at baseline give a predictive model with high significance (p<0.00002) and accuracy acc = 92%. However, the high correlation between these 2 cell counts makes this contribution less robust.
(TIF)

S9 Fig Gating strategy for identification of T-cell subsets. The figure is showing the gating strategy for the identification of CD3^+^ T-cells and its subsets CD4^+^ and CD8^+^ T-cells. EM = Effector Memory, CM = Central Memory, Eff = Effector, N = Naïve, RTE = Recent Thymic Emigrants.
(TIF)

S10 Fig Gating strategy for identification of B-cell subsets. The figure is showing the gating strategy for the identification of CD19^+^ B-cells and its subsets. M = Memory, N = Naive.
(TIF)

S11 Fig Gating strategy for identification of Dendritic cells. The figure is showing the gating strategy for the identification of Dendritic cell populations. C = Classical Monocytes, NC—Non-classical Monocytes, T = Transitional Monocytes, pDC = plasmacytoide dendritic cells, mDC = myeloide dendritic cells.
(TIF)

S12 Fig Gating strategy for identification of regulatory T-cell populations. The figure is showing the gating strategy for the identification of regulatory T-cell populations. nTreg = natural regulatory T-cells, N = Naïve, M = Memory.
(TIF)

S13 Fig Gating strategy for identification of Natural Killer cells (NK). The figure is showing the gating strategy for the identification of Natural Killer cells.
(TIF)

S14 Fig Gating strategy for identification of influenza specific CD4^+^CD40L^+^ cells. The figure is showing the gating strategy for the identification of influenza specific CD4^+^CD40L^+^ cells.
(TIF)

S15 Fig Gating strategy for identification of Plasmablasts. The figure is showing the gating strategy for the identification of Plasmablasts.
(TIF)

S16 Fig Gating strategy for identification of proliferating CD4^+^ and CD8^+^ cells. The figure is showing the gating strategy for the identification of CD4^+^Ki67^+^ and CD8^+^Ki67^+^ cells.
(TIF)

S1 Table Pilot study data. The table includes all data which were used to do the analysis presented in the paper for the pilot study. The first three columns contain the ids, the age (in years) and the gender (F-female, M-male) of the donors. The following six columns show the HAI titer for each virus strain prior to (day0) and after (day21) vaccination. Please note, that HAI titers below 10 were set to 2. The next columns show the definition of the donors as either sero-negative (HAI titer <10, indicated as 1) before and as still non-protected (HAI titer <40, indicated as 1) after vaccination. The remaining columns contain the counts per microliter for each of the immune cell population shown in Fig 2 (labels are the same as in Fig 2).
(XLSX)

S2 Table Validation study data. The table includes all data which were used to do the analysis presented in the paper for the validation study. The first three columns contain the ids, the age (in years) and the gender (F-female, M-male) of the donors. The following six columns show the HAI titer for each virus strain prior to (day0) and after (day21) vaccination. Please note, that HAI titers below 10 were set to 2. The next columns show the definition of the donors as either sero-negative (HAI titer <10, indicated as 1) before and as still non-protected (HAI titer <40, indicated as 1) after vaccination. The remaining columns contain the counts per microliter for the immune cell population measured in the validation study.
(XLSX)
