# Supplementary figures and images for: Highly Predictive Model for a Protective Immune Response to the A(H1N1)pdm2009 Influenza Strain after Seasonal Vaccination
Source: PLoS One. 2016 Mar 8;11(3):e0150812. doi: 10.1371/journal.pone.0150812 (PMC4782986; doi:10.1371/journal.pone.0150812)

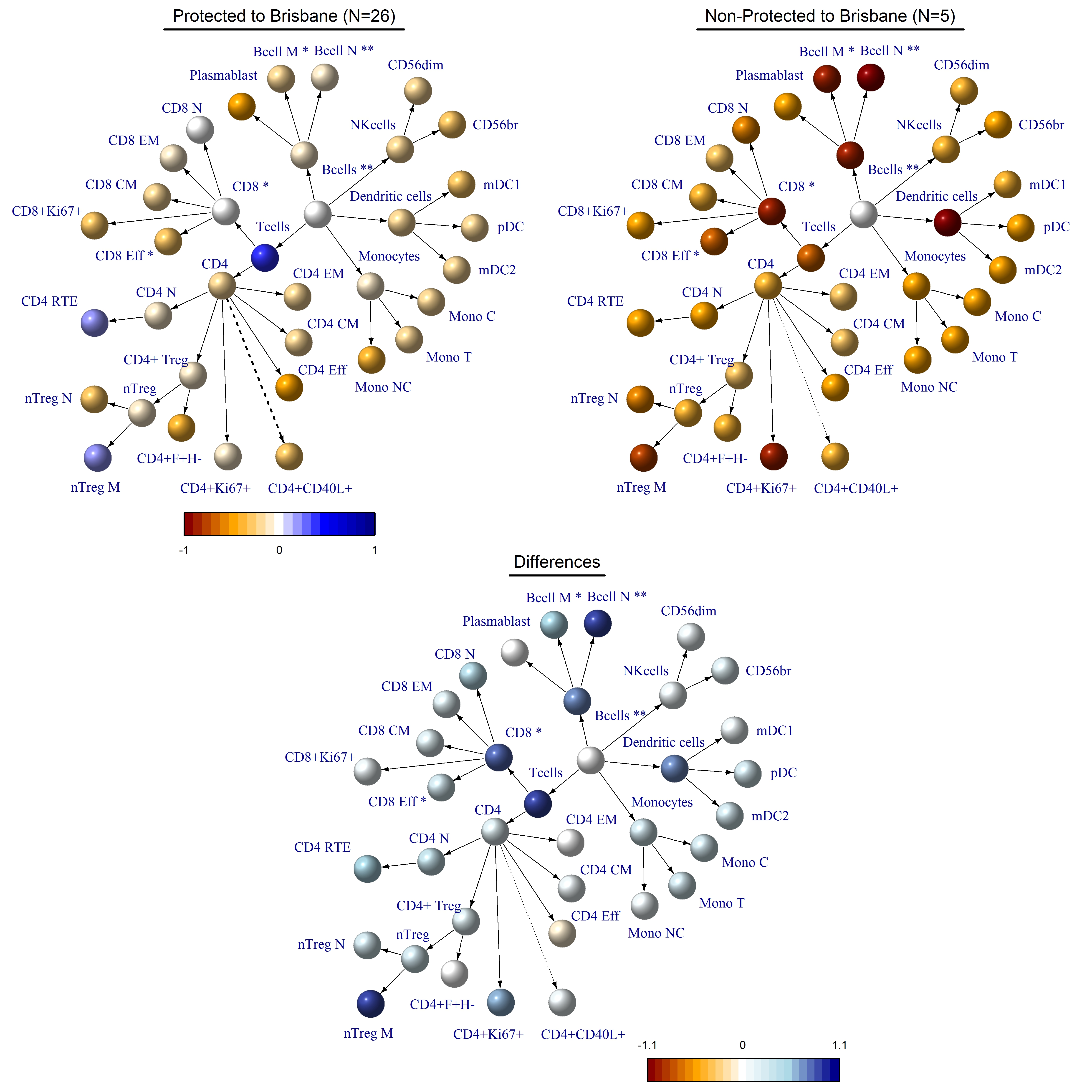

Supplement: S1 Fig — Overall, 36 immune cell subsets at baseline were analyzed and compared in Brisbane sero-negative donors with or without sero-protection for the Brisbane strain after vaccination using the Wilcoxon test. P-values below 0.05 and below 0.01 were indicated with one or two asterisks, respectively. (TIF) [file pone.0150812.s001.tif]

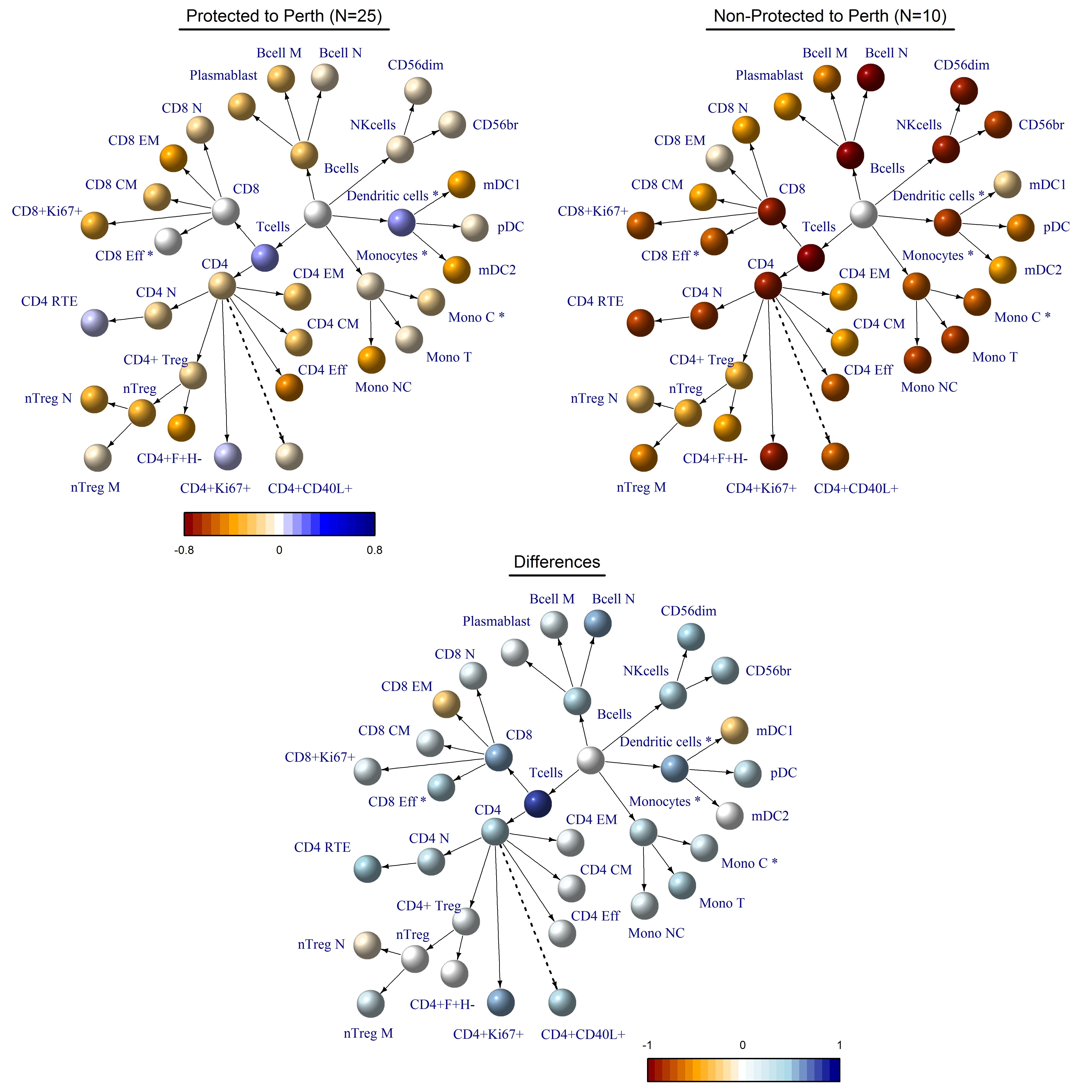

Supplement: S2 Fig — Overall, 36 immune cell subsets at baseline were analyzed and compared in Perth sero-negative donors with or without sero-protection for the Perth strain after vaccination using the Wilcoxon test. P-values below 0.05 and below 0.01 were indicated with one or two asterisks, respectively. (TIF) [file pone.0150812.s002.tif]

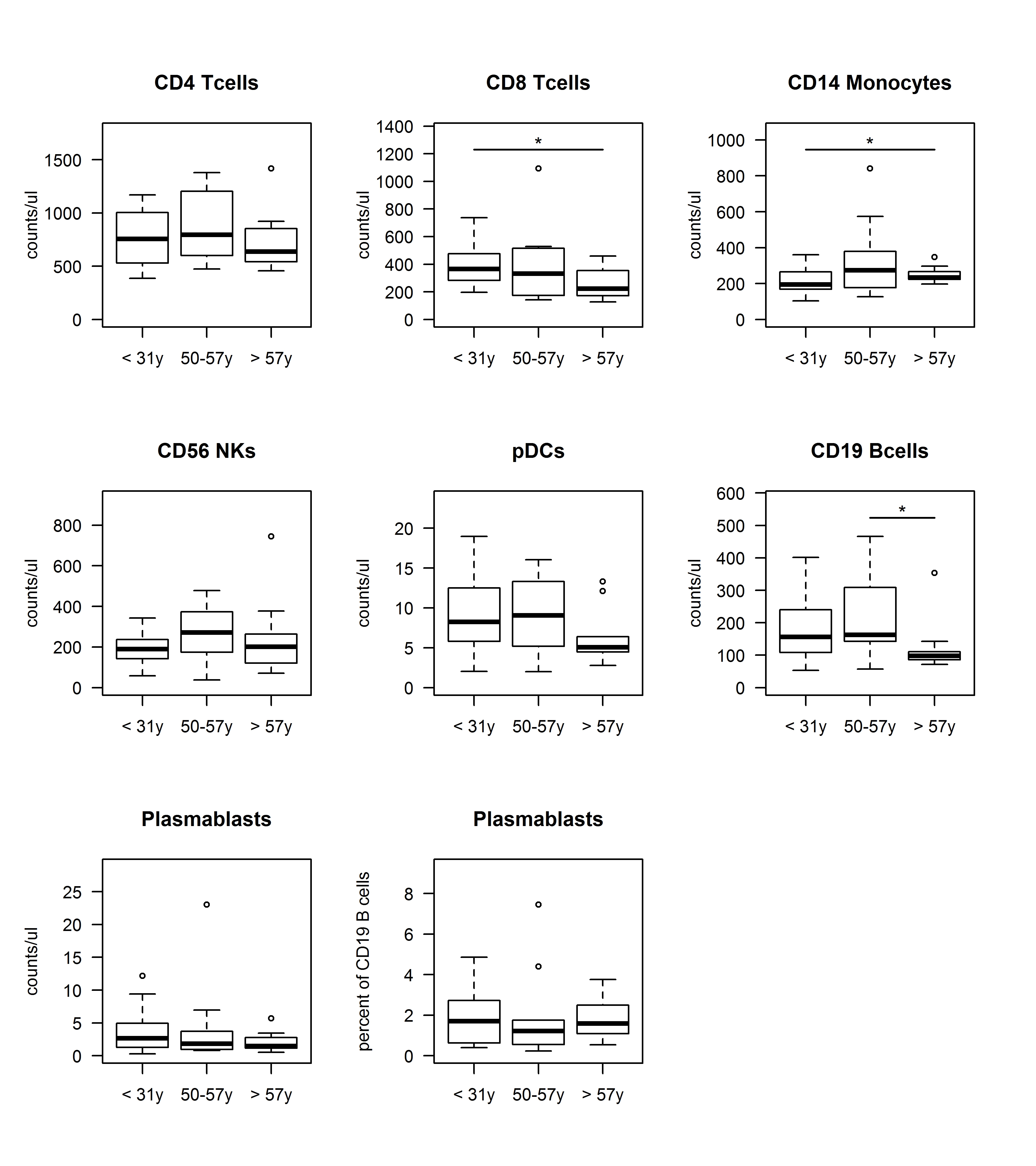

Supplement: S3 Fig — The counts of CD8+ T cells and Monocytes are significantly different between young and old donors (two-sided Wilcoxon test, p<0.05). (TIF) [file pone.0150812.s003.tif]

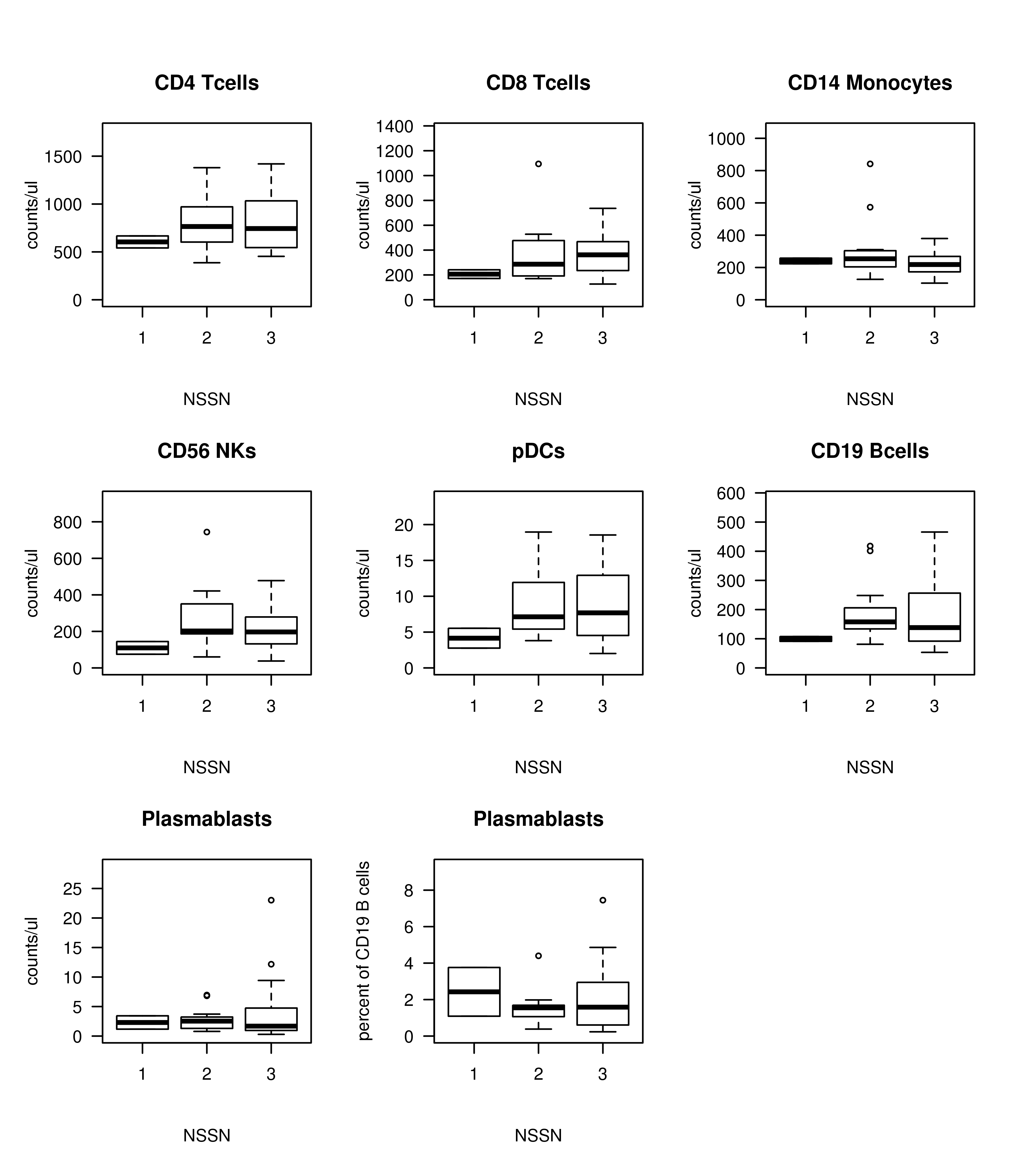

Supplement: S4 Fig — No significant differences related to the number of sero-negative strains were observed in A(H1N1)pdm09 sero-negative donors. (TIF) [file pone.0150812.s004.tif]

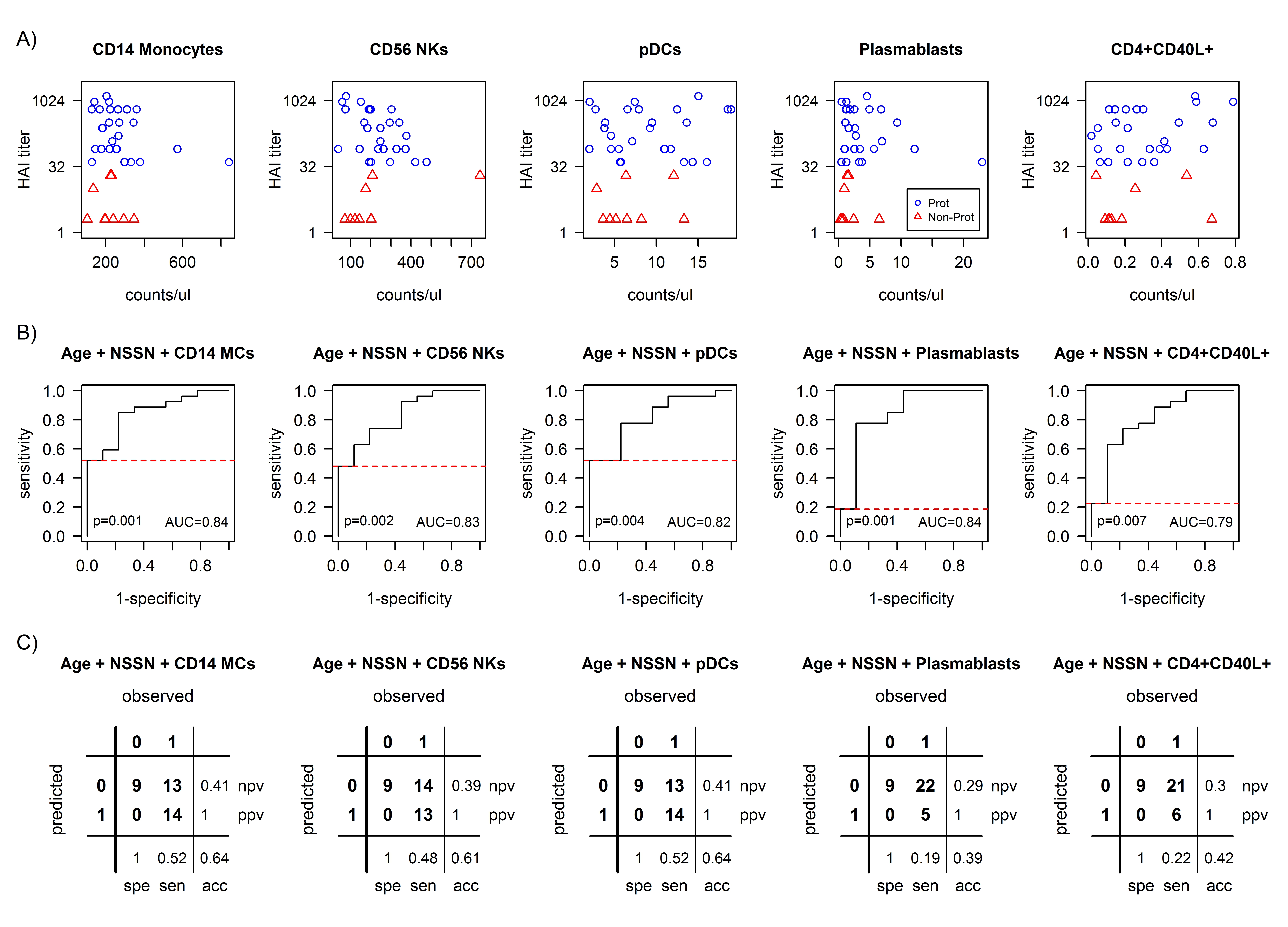

Supplement: S5 Fig — Plasmablasts counts are significantly (p = 0.03) different between H1N1 protected and non-protected vaccinees, while the other sub-populations in this figure show no significant association with protection on their own. The prediction of serological response using multi-variate logistic regression including baseline immune cell populations, age and NSSN is presented. Even if significant, the results for these cell populations are not as good as the CD4+ T cell model. In particular of interest that the model using baseline counts of specifically activated cells (CD4+CD40L+) sorted after stimulation with the 3 influenza strains in the vaccine does not give a good prediction. (TIF) [file pone.0150812.s005.tif]

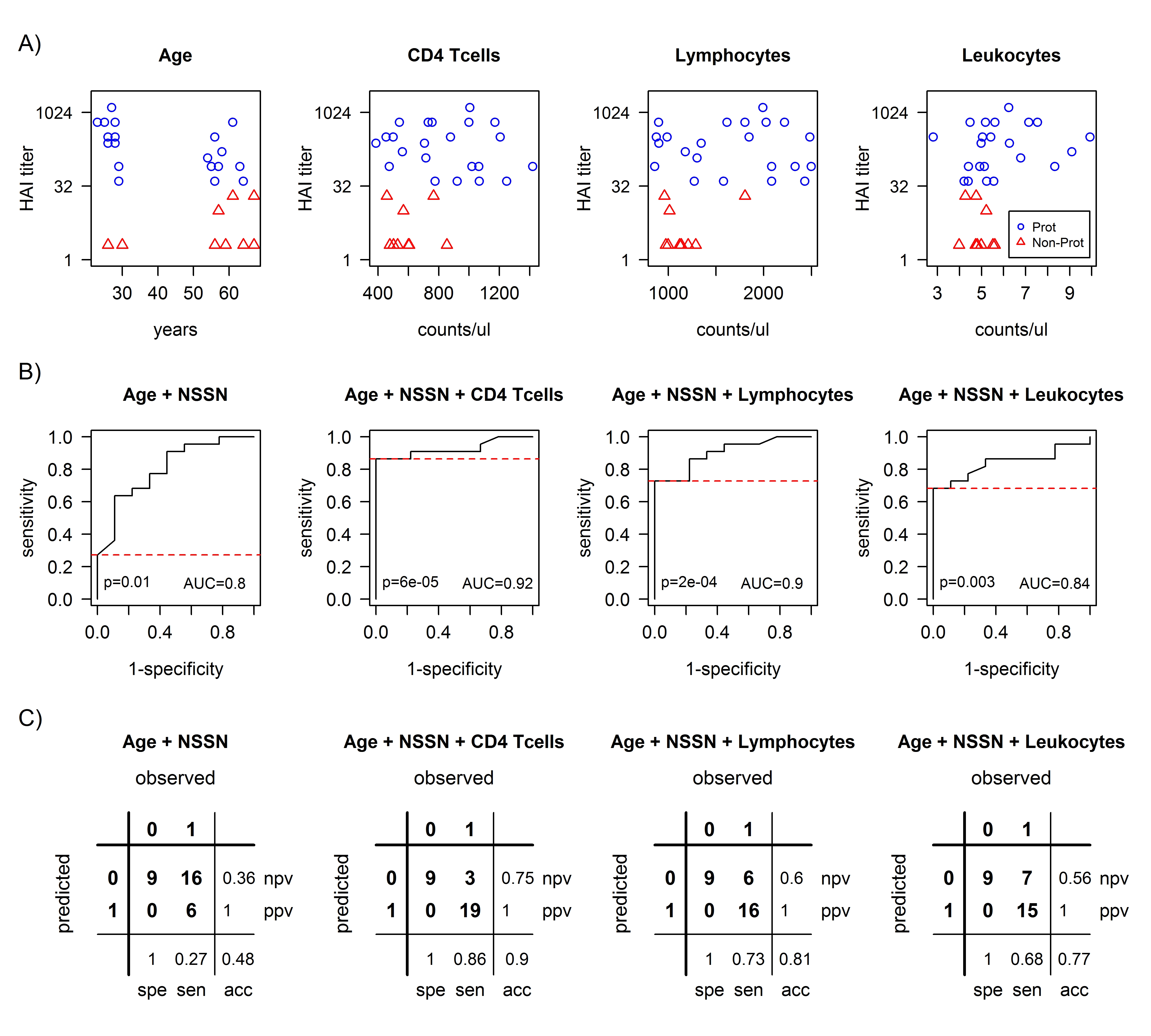

Supplement: S6 Fig — Age, CD4 T-cell and lymphocyte counts are significantly (p = 0.01, p = 0.04 and p = 0.05, respectively) different between H1N1 protected and non-protected vaccinees, while leukocytes show no significant association with protection on their own. Leukocytes and lymphocytes multivariate logistic regression models (including age and NSSN) are not as good as the CD4+ T cell multivariate logistic regression model. Due to limited availability of leukocyte data this analysis was performed in only N = 31 California sero-negative donors. Also the model using age and NSSN alone, without CD4+ T cell counts, does not give a good prediction. (TIF) [file pone.0150812.s006.tif]

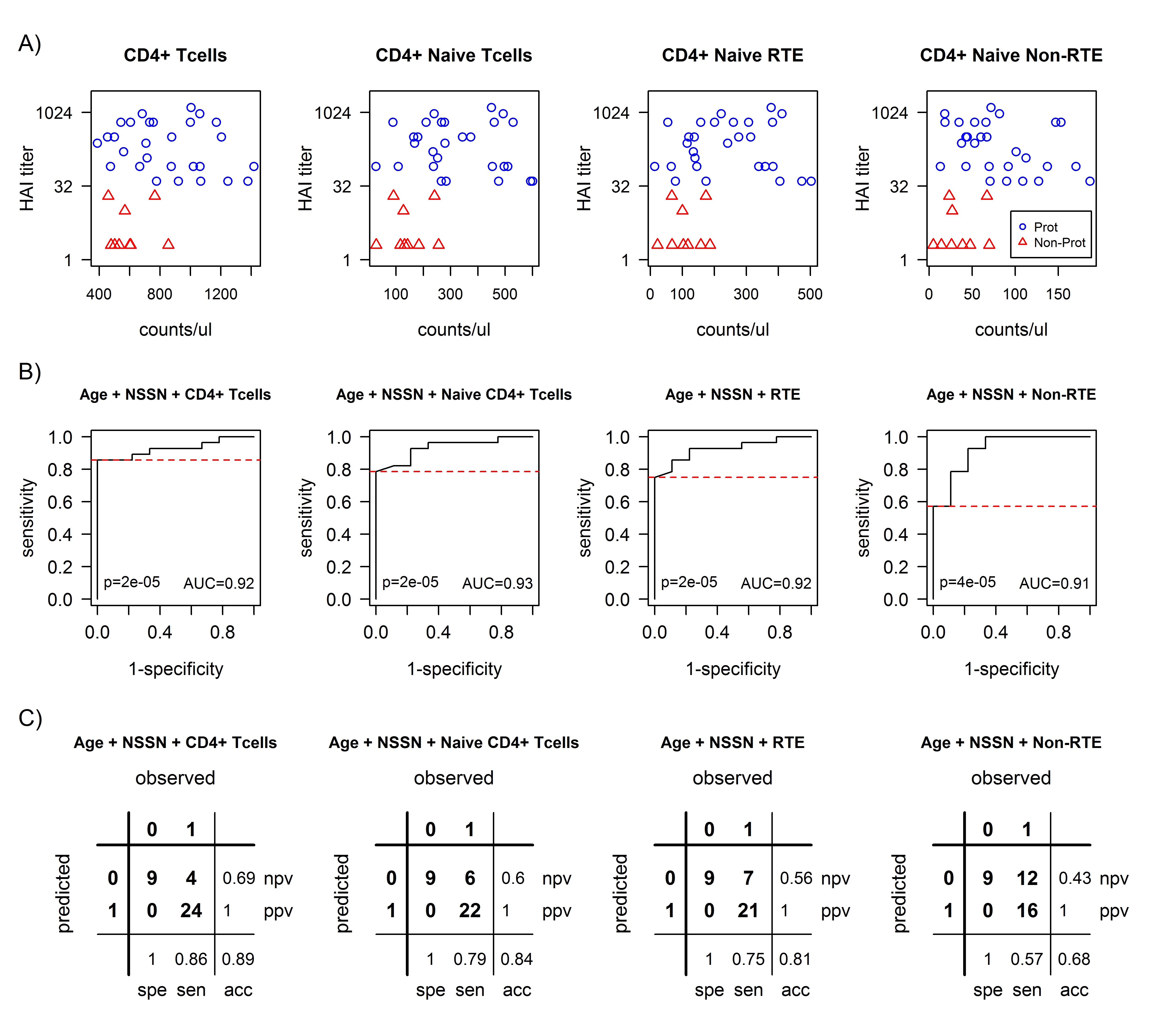

Supplement: S7 Fig — Counts of CD4 total T-cells, CD4 Naive T-cells, CD4 Naïve RTE T-cells and CD4 Naïve non-RTE T-cells are all significantly (p = 0.02, p = 0.002, p = 0.009 and p = 0.005, respectively) different between H1N1 protected and non-protected vaccinees. Prediction of serological response using multi-variate logistic regression including baseline immune cell populations, age and NSSN is presented. The prediction using the naive CD4+ T cells, RTE or non-RTE cells, is significant albeit less accurate than using the total CD4+ T cells. (TIF) [file pone.0150812.s007.tif]

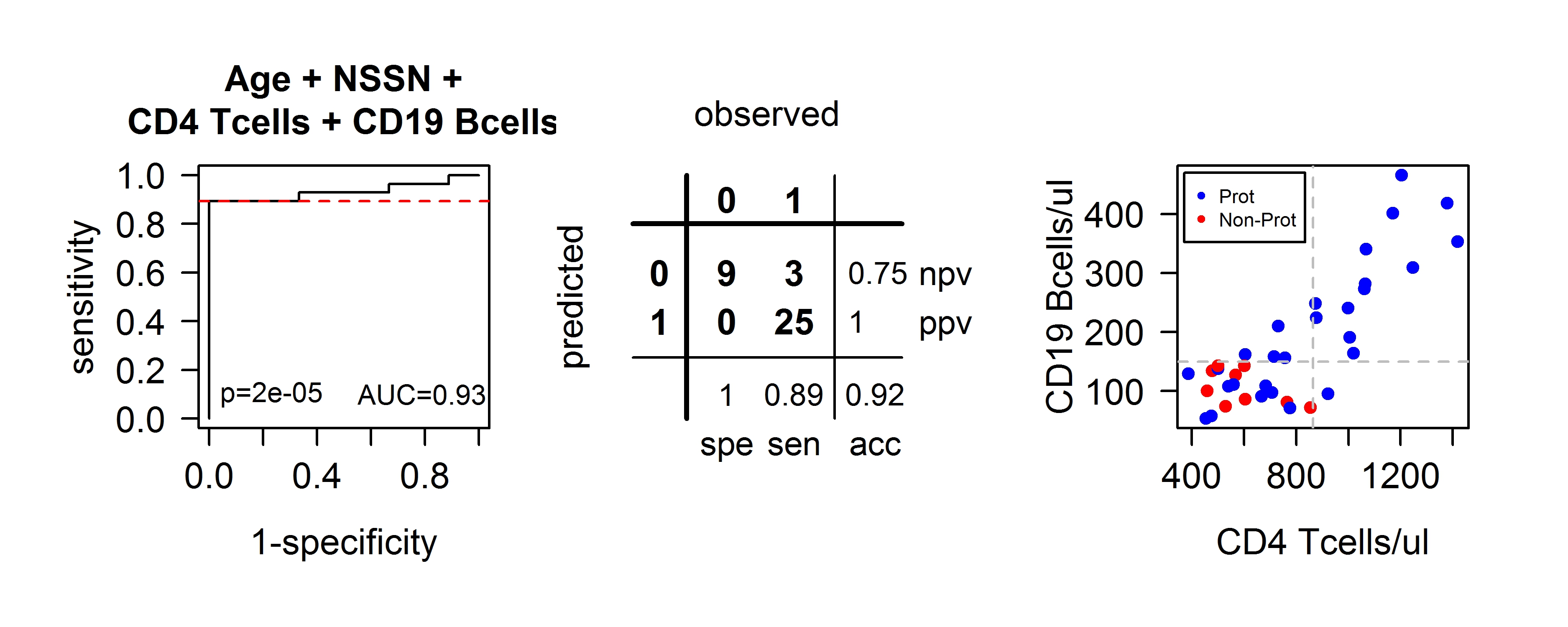

Supplement: S8 Fig — Using multi-variate logistic regression shows that the combination of age, number of sero-negative strains (NSSN), CD4+ T-cell counts and B-cell counts at baseline give a predictive model with high significance (p<0.00002) and accuracy acc = 92%. However, the high correlation between these 2 cell counts makes this contribution less robust. (TIF) [file pone.0150812.s008.tif]

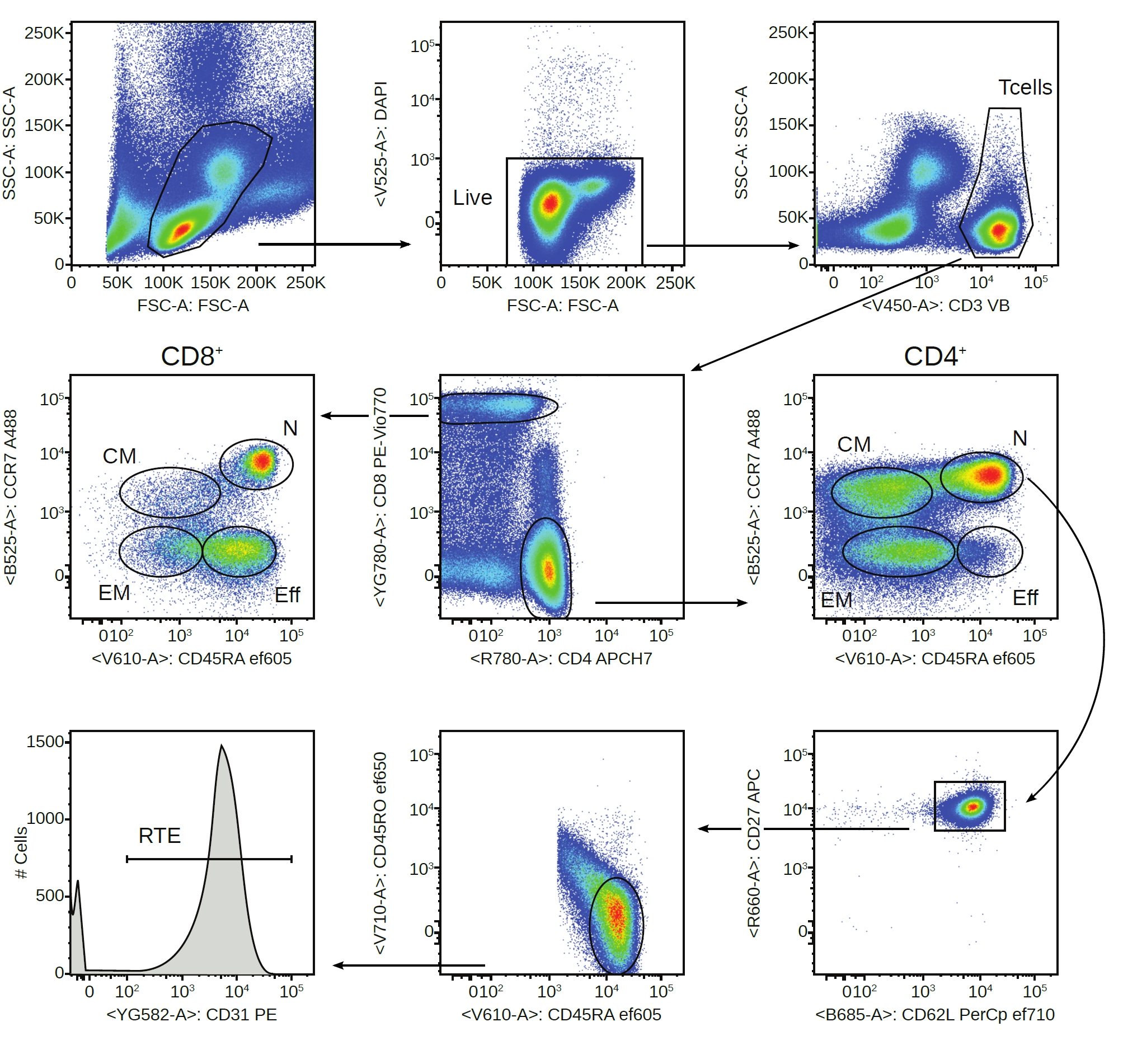

Supplement: S9 Fig — The figure is showing the gating strategy for the identification of CD3+ T-cells and its subsets CD4+ and CD8+ T-cells. EM = Effector Memory, CM = Central Memory, Eff = Effector, N = Naïve, RTE = Recent Thymic Emigrants. (TIF) [file pone.0150812.s009.tif]

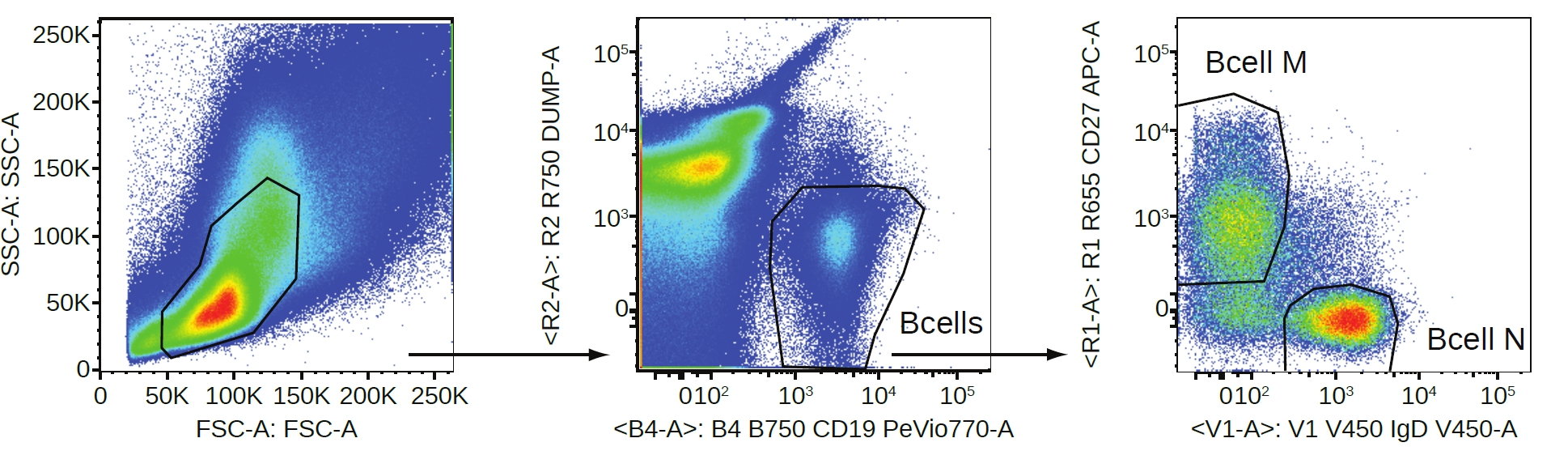

Supplement: S10 Fig — The figure is showing the gating strategy for the identification of CD19+ B-cells and its subsets. M = Memory, N = Naive. (TIF) [file pone.0150812.s010.tif]

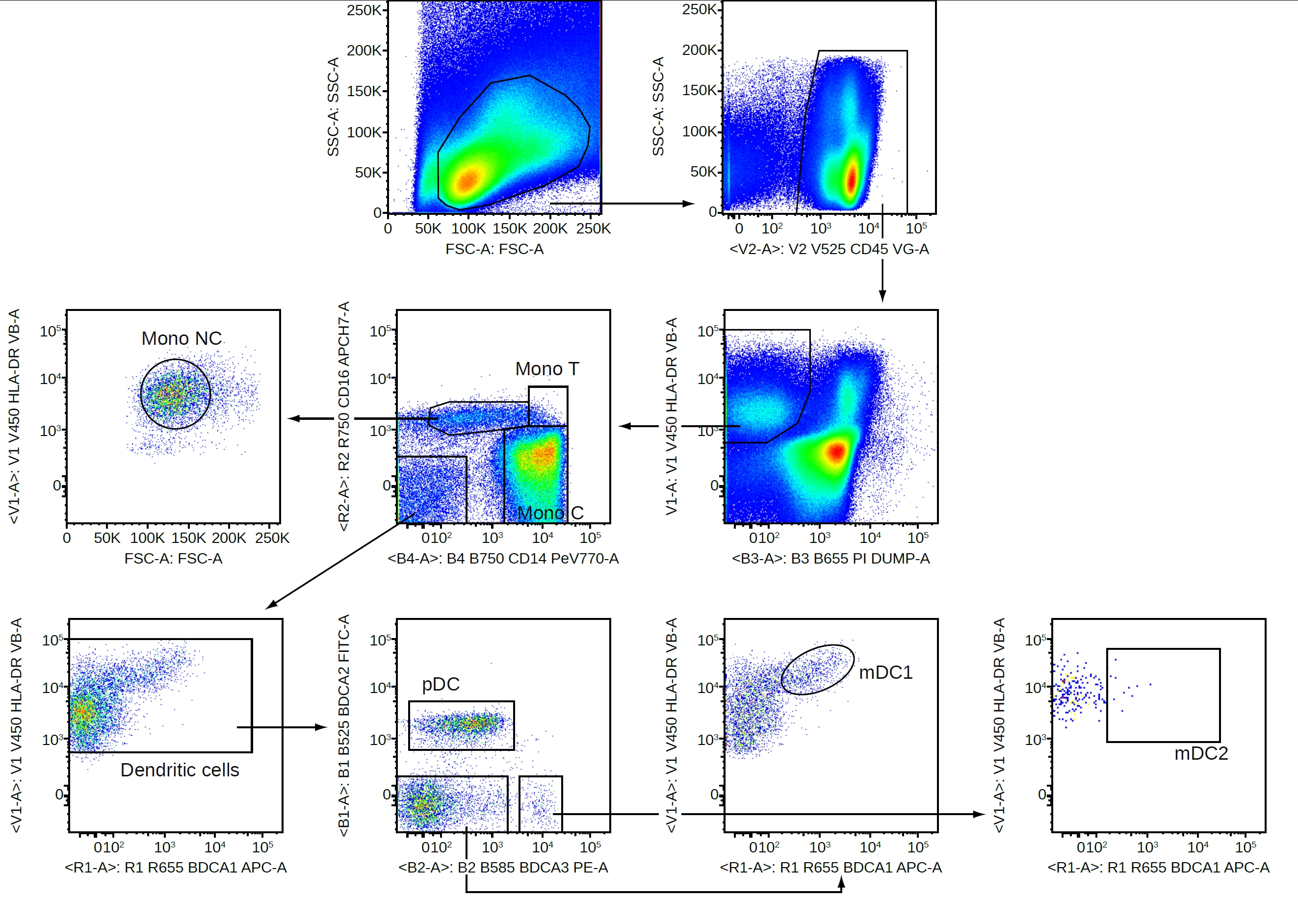

Supplement: S11 Fig — The figure is showing the gating strategy for the identification of Dendritic cell populations. C = Classical Monocytes, NC—Non-classical Monocytes, T = Transitional Monocytes, pDC = plasmacytoide dendritic cells, mDC = myeloide dendritic cells. (TIF) [file pone.0150812.s011.tif]

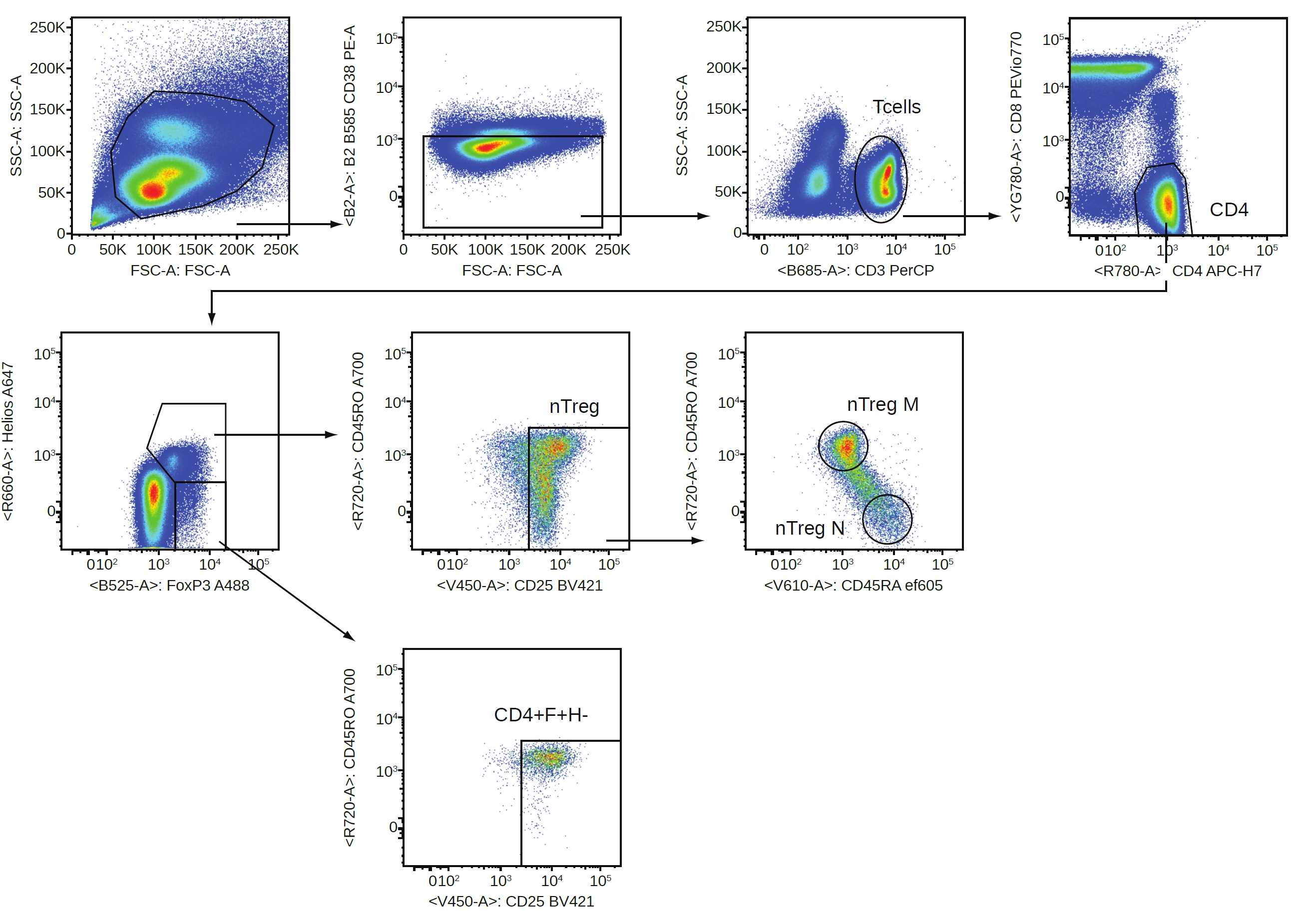

Supplement: S12 Fig — The figure is showing the gating strategy for the identification of regulatory T-cell populations. nTreg = natural regulatory T-cells, N = Naïve, M = Memory. (TIF) [file pone.0150812.s012.tif]

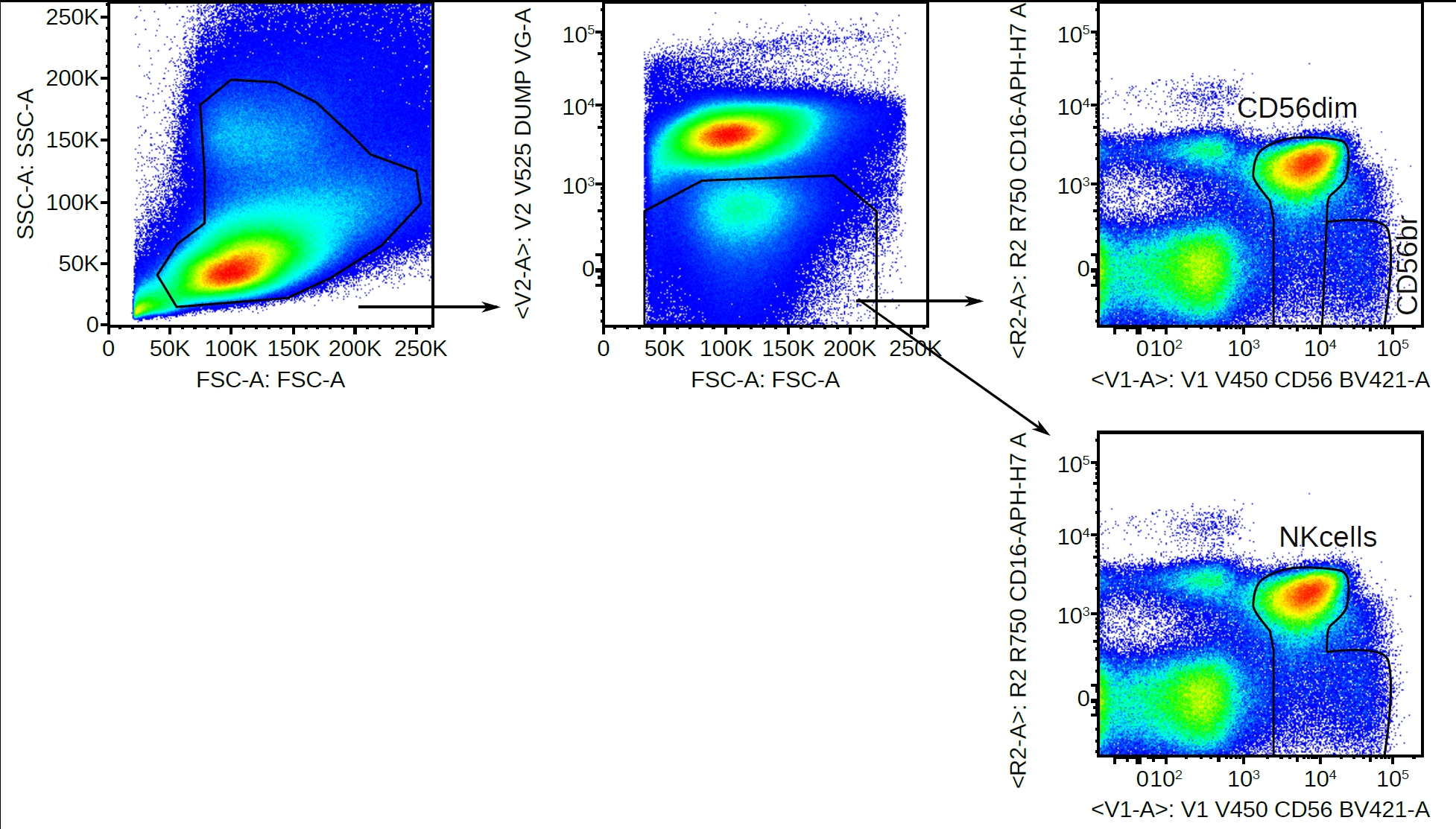

Supplement: S13 Fig — The figure is showing the gating strategy for the identification of Natural Killer cells. (TIF) [file pone.0150812.s013.tif]

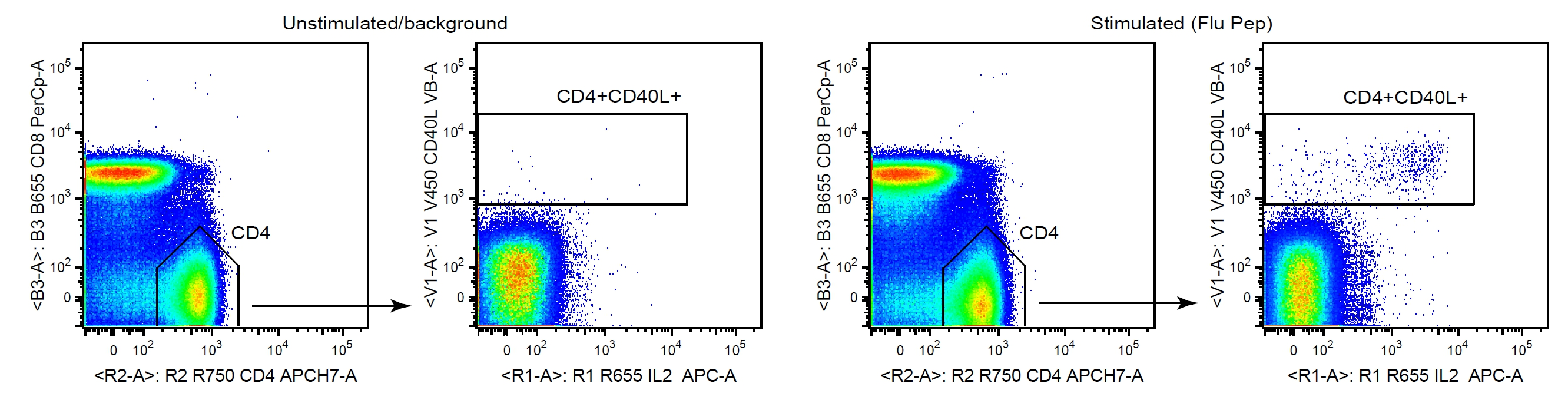

Supplement: S14 Fig — The figure is showing the gating strategy for the identification of influenza specific CD4+CD40L+ cells. (TIF) [file pone.0150812.s014.tif]

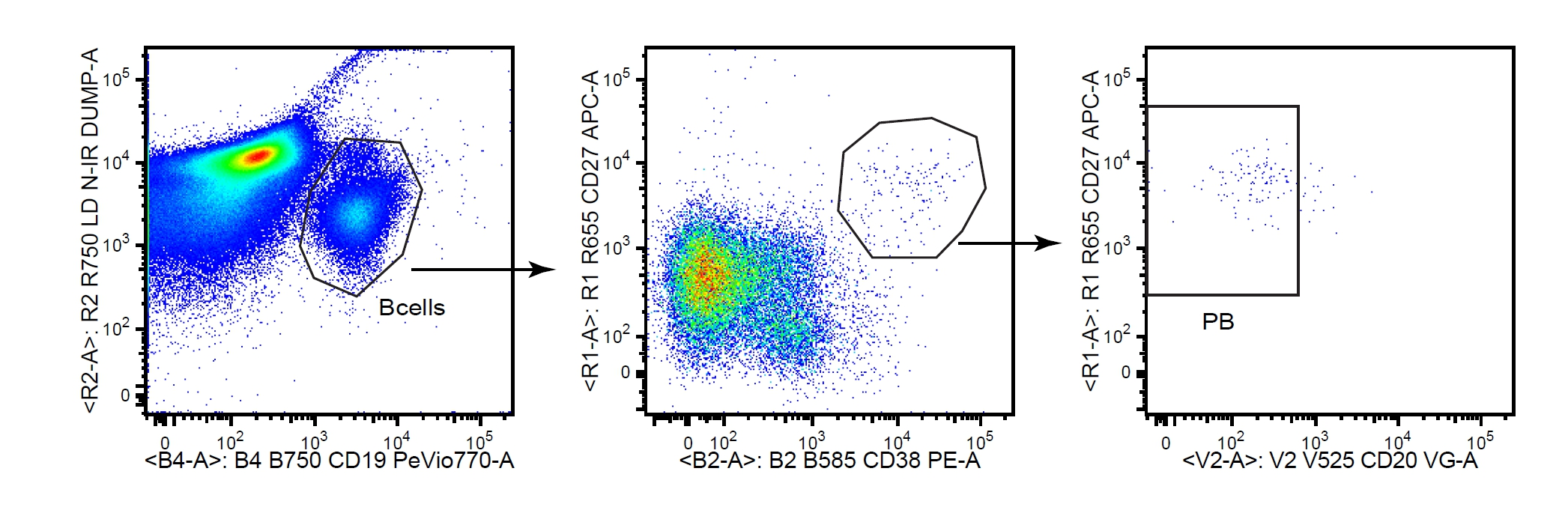

Supplement: S15 Fig — The figure is showing the gating strategy for the identification of Plasmablasts. (TIF) [file pone.0150812.s015.tif]

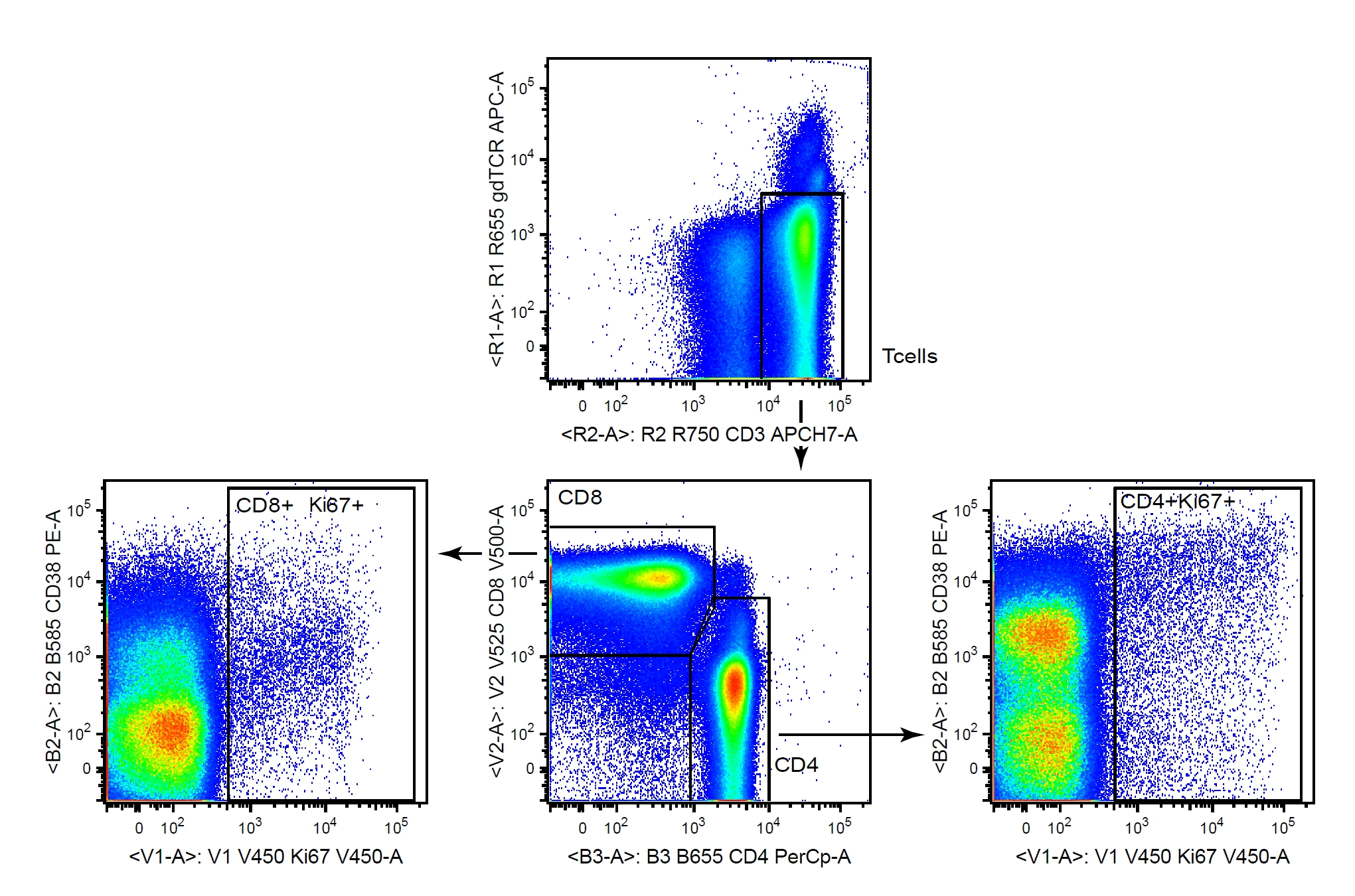

Supplement: S16 Fig — The figure is showing the gating strategy for the identification of CD4+Ki67+ and CD8+Ki67+ cells. (TIF) [file pone.0150812.s016.tif]
